# The Prognostic Quality of Risk Prediction Models to Assess the Individual Breast Cancer Risk in Women: An Overview of Reviews

**DOI:** 10.1155/2024/1711696

**Published:** 2024-03-21

**Authors:** Sarah Wolf, Ingrid Zechmeister-Koss, Irmgard Fruehwirth

**Affiliations:** HTA Austria-Austrian Institute for Health Technology Assessment (AIHTA) GmbH, Garnisongasse 7/21, Vienna 1090, Austria

## Abstract

**Purpose:**

Breast cancer is the most common cancer among women globally, with an incidence of approximately two million cases in 2018. Organised age-based breast cancer screening programs were established worldwide to detect breast cancer earlier and to reduce mortality. Currently, there is substantial anticipation regarding risk-adjusted screening programs, considering various risk factors in addition to age. The present study investigated the discriminatory accuracy of breast cancer risk prediction models and whether they suit risk-based screening programs.

**Methods:**

Following the PICO scheme, we conducted an overview of reviews and systematically searched four databases. All methodological steps, including the literature selection, data extraction and synthesis, and the quality appraisal were conducted following the 4-eyes principle. For the quality assessment, the AMSTAR 2 tool was used.

**Results:**

We included eight systematic reviews out of 833 hits based on the prespecified inclusion criteria. The eight systematic reviews comprised ninety-nine primary studies that were also considered for the data analysis. Three systematic reviews were assessed as having a high risk of bias, while the others were rated with a moderate or low risk of bias. Most identified breast cancer risk prediction models showed a low prognostic quality. Adding breast density and genetic information as risk factors only moderately improved the models' discriminatory accuracy.

**Conclusion:**

All breast cancer risk prediction models published to date show a limited ability to predict the individual breast cancer risk in women. Hence, it is too early to implement them in national breast cancer screening programs. Relevant randomised controlled trials about the benefit-harm ratio of risk-adjusted breast cancer screening programs compared to conventional age-based programs need to be awaited.

## 1. Introduction

Breast cancer is the most common cancer among women globally, with an incidence of approximately two million cases worldwide in 2018 [1]. In high-income countries, about 75% of breast cancer cases are diagnosed in postmenopausal women and only five–seven % affect women younger than 40 [2, 3]. The illness exhibits heterogeneity, encompassing various histological and molecular subtypes stemming from diverse aetiologies, each exhibiting differential responses to treatment and prognosis [4, 5]. Factors such as increasing age, high breast density, history of neoplastic breast disease, family history of breast cancer, genetic predispositions (single nucleotide polymorphisms (SNPs)) (single nucleotide polymorphisms are variations of a single base pair in a complementary DNA double strand and are inherited and heritable genetic variants), as well as hormonal, lifestyle, or radiation exposure factors, can increase the risk of developing breast cancer [6–11].  Table 1 presents the criteria usually considered to identify women with an increased risk of developing breast cancer.

To date, great hope is placed in a risk-based screening approach. Since the early 1970s, organised breast cancer mammography screening programs have been established worldwide to reduce mortality by earlier cancer diagnosis [12–15]. The only risk factor considered so far in these programs is age. In risk-based screening, risk prediction models estimate the likelihood of women developing breast cancer in the future, considering other risk factors next to age [16–18]. By considering multiple risk factors, women could be stratified into different risk groups, which enable risk-adjusted screening strategies. For example, less frequent mammograms could be recommended for women with a low risk of breast cancer. Hence, risk-adjusted breast cancer screening might reduce the disadvantages of conventional age-based screening programs, e.g., overdiagnosis and overtreatment, or enable breast cancer diagnosis at an earlier stage [19–23].

There are empirical, genetic, and other original risk prediction models. Empirical models, e.g., the Gail model (the Breast Cancer Risk Assessment Tool (BRCAT)), the Breast Cancer Surveillance Consortium (BSCS) model, and the Rosner–Colditz model include risk factors previously identified by logistic regression and Cox proportional hazard regression in cohort and case-control studies [24]. Using a statistical algorithm, these models generate the probability that an individual will develop breast cancer in a given time [24]. Genetic models, e.g., the International Breast Cancer Intervention Study (IBIS)/Tyrer–Cuzick model and the BOADICEA and BRCAPRO™ models, are based on the evaluation of family studies and segregation analyses. In addition, pedigree information is used to calculate age-dependent mutation and disease risks for all family members [25]. Tables S1a and S1b of the supplement provides an overview of the characteristics of the most common empirical and genetic breast cancer prediction models, including a list of risk factors considered in each model. Besides, some further original models combine various risk factors in different populations with different algorithms, e.g., the Barlow model [26] for pre- and postmenopausal women.

Our study aimed to investigate the prognostic quality of the identified breast cancer risk prediction models and whether they are suitable for assessing individual breast cancer risk in a screening program.

## 2. Materials and Methods

We conducted an overview of reviews, considering most of the Preferred Reporting Items for Overviews of Reviews (PRIOR) statement [27]. An overview of reviews was the appropriate methodological approach because a preliminary search yielded several published systematic reviews (SRs) regarding the prognostic quality of individual breast cancer risk prediction models. Thereby, the extensive knowledge from the SRs could be summarised as concisely as possible.

### 2.1. Literature Searches

In March 2022, we conducted a comprehensive systematic literature search in four databases, namely, Ovid MEDLINE, EMBASE, the Cochrane Library, and CRD. The systematic literature search was performed considering the predefined inclusion criteria according to the PICO scheme (Table 2). The detailed search strategy is presented in the supplement (see Tables S2–S6).

In addition, we conducted further manual searches to identify the full texts of the primary studies of the selected SRs for more detailed information if relevant.

### 2.2. Literature Selection Process

The systematic literature search yielded references initially assessed at the title level. Subsequently, references deemed pertinent underwent screening at the abstract level. Finally, full texts of relevant abstracts were scrutinised against predefined inclusion criteria for incorporation or exclusion in the overview of reviews. Two reviewers (IF and SW) conducted all procedures independently, with discrepancies resolved through discussion involving a third author (IZK).

### 2.3. Assessed Primary Outcome

The primary effectiveness outcome of this overview of reviews was the discriminatory accuracy of the identified breast cancer risk prediction models; that is to say, the probability that a model correctly categorises a randomly chosen woman with the disease at higher risk than a randomly chosen woman without the disease. To provide the most accurate individual risk assessment, the models need to balance the diagnostic sensitivity and specificity represented by the receiver operating characteristic curve (ROC). The area under this curve (AUC) quantifies the discriminatory accuracy of a prediction model. An AUC value of 0.5 indicates that the discriminatory accuracy of a model is no better than a coin toss. In contrast, an AUC value of 1.0 denotes perfect discriminatory accuracy. In practice, models with an AUC value greater than 0.7 are deemed to predict the individual risk for breast cancer at acceptable accuracy.

### 2.4. Data Extraction and Quality Appraisal

One author (IF) extracted the characteristics of the included SRs and the data of the SRs on primary study level. IF extracted further data directly from the primary studies if necessary information was missing. A second author (SW) controlled the data extraction. Both authors (IF and SW) assessed the quality of the selected SRs independently according to the AMSTAR 2 tool. The checklist encompasses inquiries about the methodological procedures employed in a review, the thoroughness of the results and conclusions, the origins of funding, and the presence of potential conflicts of interest [28]. The overall risk of bias of the systematic reviews included in this overview was evaluated independently by two authors (IF and SW) through a comparative analysis of the checklist findings derived from the included reviews. Differences were discussed and resolved by consensus of all three authors (IF, SW, and IZK).

### 2.5. Analysis and Synthesis

Finally, we narratively summarised the evidence on the prognostic quality of the identified prediction models, including two tables that present the key results. The detailed extraction tables showing the data on the primary study level are presented online.

## 3. Results

### 3.1. Literature Selection

**Figure 1:**
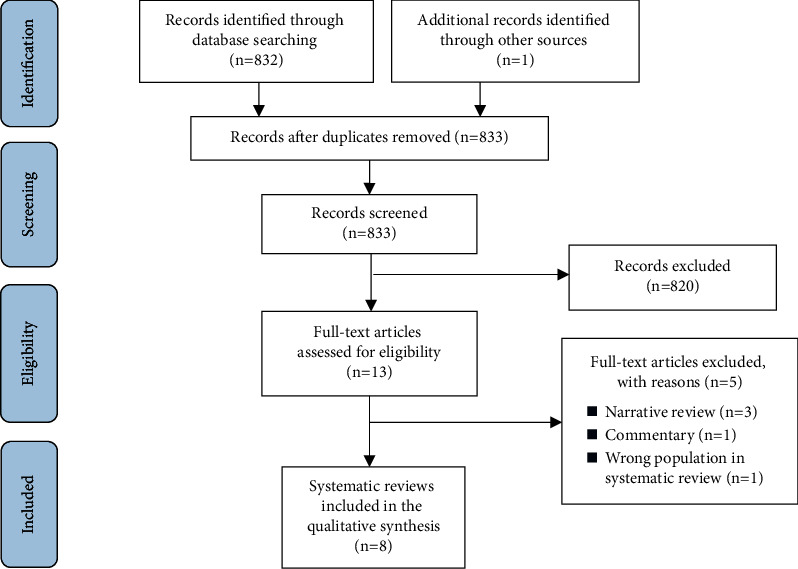
Representation of the literature selection process (PRISMA flow diagram).

The systematic literature search and additional manual searches yielded 833 references. Out of the 833 hits, we included eight SRs based on the predefined inclusion criteria (Table 2) [16, 29, 30, 48–52]. The detailed literature selection process with reasons for exclusion is illustrated in Figure 1.

### 3.2. Characteristics of the Systematic Reviews

The eight included SRs were written in English and published between 2012 [48] and 2020 [52]. Based on the affiliations of the first authors, five SRs were from Europe (the UK [16, 49], Denmark [52], the Netherlands [29], and Spain [30]). The remaining three SRs were from the Asian region (Thailand [48], China [50], and Singapore [51]).

The eight SRs included 99 studies (between 12 and 63 per SR) published from 1989 to 2019. Often, the same studies were included in multiple reviews. One SR [48] only reported data from 18 of 25 included studies. The remaining seven studies were not described in detail. Seven of the eight SRs reported the study design of the included studies. The predominant study designs were case-control and cohort studies. Of the eight SRs, six included various ethnicities, among them Caucasian/White [29, 30, 48, 49, 51, 52], Asian [30, 48, 49, 51, 52], African-American [29, 30, 48, 51], Hispanic [30, 49, 51], African [49], and Australian [51]. Two SRs [16, 50] did not report which populations were included in the studies assessed.

The SRs investigated 30 risk prediction model versions (between one and 17 per SR) with different research focuses. One SR [50] examined the performance of various Gail/BRCAT model versions. Two other SRs [51, 52] investigated the improvement in the discrimination accuracy of the models by adding essential risk factors, such as genetic information or breast density. The remaining five SRs [16, 29, 30, 48, 49] compared the model performance with each other or examined the use of multivariable prediction models in risk-based cancer screening programs. One of the five SRs [29] evaluated breast, cervical, and colorectal cancer risk prediction models. However, for this overview of reviews, only the results concerning the breast cancer risk prediction models were considered.

The primary outcome parameters in all eight SRs were the discriminatory accuracy and the calibration accuracy of the breast cancer risk prediction models. This overview of reviews focused solely on the discriminatory accuracy of the models.

Table S7 of the supplement presents the characteristics of the included SRs in more detail.

### 3.3. Quality Assessment

Two of the included SRs were rated with a low risk of bias [16, 30] and three with a moderate risk of bias [50–52]. The remaining three systematic reviews were rated with a high risk of bias [29, 48, 49]. The major flaws were due to significant methodological limitations, including unclear literature selection and data collection processes. Moreover, no quality assessment of the primary studies was performed in three SRs [29, 48, 52], while the remaining five SRs assessed the quality of the studies using different methods [16, 30, 49–51].  Table S8 of the supplement presents the quality assessment in detail.

### 3.4. Discrimination Accuracy of the Identified Breast Cancer Risk Prediction Models

#### 3.4.1. Empirical and Genetic Models

*(1) The Gail/Breast Cancer Risk Assessment Model (Empirical)*. In the eight included SRs [16, 29, 30, 48–52], 58 validation studies analysed how accurately the Gail model can predict individual breast cancer risk. 33 of the 58 validation studies were from the United States of America (USA), 12 from Asia, 10 from Europe, and 3 from Australia. Most validation studies included Caucasian/White/European populations. Besides, the studies also considered North American, Asian, Hispanic, African-American, and Australian populations. Two publications did not report on the population.

The Gail model is the most investigated and modified breast cancer risk prediction model. The original Gail model, developed in 1989, includes the following five risk factors: age, family history of breast cancer, age at first birth, age at menarche, and previous biopsies [53]. Since then, the original model has been validated in various populations (e.g., Caucasian/White/European, American, African-American, Asian, or Hispanic) and has been modified many times by adding risk factors, such as breast density or hormone replacement therapy. Regarding the prognostic quality of the Gail model 1, AUC values ranging from 0.54 [54] to 0.69 [55] were reported. Adding or removing risk factors, such as breast density, hormone replacement therapy, alcohol consumption, physical activity, diet, or ethnicity, to or from the Gail model did not improve the models' discrimination accuracy (e.g., AUC values of 0.56 [56] and 0.68 [57]). Solely a body mass index-adjusted Gail model showed an AUC value of 0.85 [52], and there were two outliers in Asian populations; one validation study showed an AUC value of 0.41 [48] and another presented a value of 0.93 [50] for the Gail model.

*(2) The Breast Cancer Surveillance Consortium Model (Empirical)*. Six validation studies, included in three SRs [16, 30, 51], assessed the prognostic quality of the BCSC model, which originates from the USA. All six validation studies included mixed ethnicities.

The original BCSC model includes the following eight risk factors: age, body mass index, age of menopause, hormone replacement therapy, breast density, prior breast biopsies, and family history of breast cancer. Concerning the prognostic quality of the original BCSC model, the validation studies showed AUC values, ranging from 0.58 to 0.67 [58]. Three validation studies added genetic information as a polygenic risk score to the model. They achieved AUC values of 0.69 [32], 0.65 [59], and 0.72 [58], whereby the latter applied to the prediction of oestrogen receptor-positive breast cancer.

*(3) The Rosner and Colditz Model (Empirical)*. In five of the eight included SRs [16, 29, 30, 48, 49], nine validation studies investigated the prognostic quality of the Rosner and Colditz model. Eight of the nine studies were from the USA, and one was from France. The nine studies considered solely Caucasian/White populations.

The original Rosner and Colditz model includes the following five risk factors: age, body mass index, hormone replacement therapy, benign breast disease, and family history of breast cancer. The original model has an AUC value of 0.57 [31] and was often modified. For example, adding serum estradiol to the model improved its discriminatory accuracy (AUC value of 0.635) [33]. Similarly, adding risk factors, such as breast density, multiple hormone level determinations, and/or a polygenic risk score, to the original Rosner and Colditz model resulted in an improved AUC value of 0.68 [60].

*(4) The International Breast Cancer Intervention Study/Tyrer-Cuzick Model (Genetic)*. Four SRs [16, 30, 51, 52] included eight validation studies on the IBIS model. Two of the eight studies came from the USA, five from the United Kingdom (UK), and one from Australia. The studies included different populations, namely, Caucasian/European, North American, African-American, Hispanic, and mixed ethnicities. One study did not report the assessed population.

The original IBIS/Tyrer–Cuzick model considers the following 14 risk factors: age, body mass index, age at menarche, age of first live birth, age of menopause, parity, hormone replacement therapy, breast density, atypical ductal hyperplasia, lobular carcinoma in situ, prior breast biopsies, family history of breast cancer (including age at diagnosis and bilateral breast cancer), family history of ovarian cancer, and genetic testing (BRCA1/2 and SNPs). The SRs and validation studies did not report an AUC value for the original IBIS/Tyrer–Cuzick model. The discriminatory accuracy of different model versions ranged from AUC values between 0.51 and 0.76, with the latter AUC value reported from a study in a high-risk European population [34–36]. IBIS/Tyrer–Cuzick model versions, including a polygenic risk score, reached an AUC value of 0.67 and versions that considered breast density as a risk factor had an AUC value of 0.64 [37].

*(5) BOADICEA and BRCAPRO™ Models (Genetic)*. One SR [51] included two validation studies that assessed the prognostic quality of two further genetic breast cancer risk prediction models. Both studies were from Australia, whereby one assessed the discriminatory accuracy of the BOADICEA model and the other of the BRCAPRO™ model. Both studies included Caucasian populations.

The original BOADICEA model includes the following six risk factors: age, family history of breast cancer with age at diagnosis, family history of male breast cancer, family history of ovarian cancer, and genetic testing (BRCA1/2 and SNPs). The BRCAPRO™ model considers two further risk factors, i.e., family history of bilateral breast cancer and ethnicity of the family. The discriminatory accuracy of the BOADICEA and the BRACAPRO™ models is moderate, with an AUC value of 0.66 and 0.65, respectively. Adding a polygenic risk score with 77 risk-associated SNPs to both models improved their discriminatory accuracy significantly with AUC values of 0.70 and 0.69, respectively [61].

Table 3 presents an overview of the discriminatory accuracy of the identified empirical and genetic breast cancer risk prediction models and shows that almost all identified model versions had a limited discriminatory accuracy with AUC values <0.70. Exceptions included a modified Gail model applied in an Asian population, a modified BCSC model that applied the prediction of oestrogen receptor-positive breast cancer, an IBIS/Tyrer–Cuzick model version applied in a high-risk European population, and the BOADICEA model expanded with SNPs.

### 3.5. Further Original Models

Six of the eight included SRs [16, 30, 48, 49, 51, 52] investigated 24 further original models. Four validation studies were from Europe, nine from the USA, nine from Asia, one from Canada, and one from India. Most validation studies included Asian populations. Besides, the studies also included Caucasian/White/European, North American, and mixed ethnicities. One study did not report on the population.

The discriminatory accuracy of these original models ranged from AUC values of 0.53 [38] to 0.785 [45], whereby the latter applied to the prediction of ER-positive, HER2-negative, invasive, and noninvasive carcinoma in a Japanese population considering a polygenic risk score. A Swedish model [46] including age, body mass index, hormone replacement therapy, family history of breast cancer, age at menopause, breast density, microcalcifications, and space-occupying lesions as risk factors showed an AUC value above 0.71 for a Caucasian population. The discriminatory accuracy of the models considering breast density as a risk factor ranged from AUC values of 0.63 [40] to 0.72 [41], depending on whether the absolute area, per cent of the area, or fibroglandular volume of breast density measurement was used. The models that included a polygenic risk score as a risk factor—except the Japanese model—had AUC values between 0.60 [38] and 0.693 [44]. The Barlow model had a moderate discriminatory accuracy with AUC values of 0.631 for premenopausal and 0.624 for postmenopausal women [26].

Table 4 summarises the discriminatory accuracy of further original breast cancer risk prediction models, depending on the considered risk factors and breast cancer types. Overall, most of the identified models have a limited discriminatory accuracy with AUC values <0.70, except a Swedish model with a two-year time horizon [46] and a Japanese model that considered SNPs [45].

In the supplement (Tables S9a–S9f), the detailed extraction tables present the data per prediction model on the primary study level.

## 4. Discussion

Most identified breast cancer risk prediction models with low prognostic quality do not accurately predict the individual breast cancer risk. Adding breast density and/or genetic information as crucial risk factors moderately improved the discriminatory accuracy of the prediction models but remained below the minimum AUC value of 0.70. Exceptions include a modified Gail model assessed in an Asian population, a modified BCSC model that applied the prediction of oestrogen receptor-positive breast cancer, an IBIS/Tyrer–Cuzick model version that was applied in a high-risk European population, the BOADICEA model that considered SNPs, and two further original models, one from Japan and one from Sweden. The AUC value above 0.70 in the Japanese study [45] may be due to the risk prediction of solely ER-positive, HER-2-negative breast cancer. The AUC value above 0.70 in the Swedish model [46] could be explained by the short time horizon of two years, as risk prediction becomes more imprecise over a longer time horizon. Overall, the differences in the AUC values can be mainly explained by differences in study populations, comprising various geographical regions, cancer risk groups, and cancer types.

Besides the discriminatory accuracy of the risk prediction models, further aspects need to be considered if these models are to be used more widely.

The identified breast cancer risk prediction models were developed and validated for use in a clinical (genetic) setting and/or to identify specific patient groups eligible for preventive intervention but not for population-based screening [47]. For example, the Gail/BRCAT model is considered suitable for identifying women who would benefit from chemoprevention [39, 42]. Therefore, the appropriate setting needs to be assessed before applying a risk prediction model.

Critical risk factors, such as breast density, come with assessment requirements. Density-based risk calculations are often based on visual density estimates using BI-RADS categories. However, objective criteria for a standardised density measurement according to BI-RADS categories are lacking in practice [43]. Volumetric density measurements are fully automated and have excellent agreement with 3D magnetic resonance images but are less informative than the BI-RADS categories [62–64]. Hence, considering breast density as a risk factor for predicting individual breast cancer risk requires a standardised density measurement. Similarly, assessing genetic information as an additional risk factor requires the organisation of cooperations between qualified centres for medical genetics.

Moreover, risk-based breast cancer screening requires valid risk prediction instruments with good prognostic quality and risk-adjusted screening strategies. Solely conducting risk assessments is not enough. Instead, low, medium, and high breast cancer risk groups need to be defined to provide women with risk-adjusted strategies where the screening intensity matches the individual risk. However, matching is only good if the applied risk assessment model has good discriminatory accuracy [65, 66]. Currently, there are no internationally uniform cutoff values for the assignment to the risk group [25].

Besides, training in risk communication is necessary for healthcare professionals when risk-adjusted screening is planned to be implemented because risk-based screening is more complex for healthcare professionals and participants than standardised age-based screening. Risk-based screening includes performing risk assessments, appropriately communicating risk results, and consulting subsequent preventive interventions. The latter, in turn, alters the risk of developing breast cancer.

From a scientific point of view, evidence is lacking on the overall benefit-harm ratio of risk-based breast cancer screening compared to conventional age-based screening programs. Therefore, the results of two large ongoing randomised control trials (RCTs) on the efficacy of risk-based breast cancer screening need to be awaited, with results expected in a few years [67, 68].

To our knowledge, this is the first overview of reviews assessing the prognostic quality of breast cancer risk prediction models and whether they apply to a population-based screening. However, the results of this overview should be viewed in the context of its limitations.

While adhering to most methodological steps outlined by the PRIOR checklist for systematic review overviews, we did not perform sensitivity analysis to assess the robustness of the review findings. In addition, although we provided results at the primary study level, we evaluated the risk of bias solely for the systematic reviews rather than for all 99 primary studies. Finally, we did not examine reporting bias in the primary studies or the systematic reviews.

Despite the inclusion of systematic reviews exhibiting varying degrees of methodological rigour, our analysis indicates that reviews with low or moderate risk of bias arrive at similar conclusions to those with a high risk of bias.

Furthermore, the selected SRs included validation studies published until 2019. Hence, the studies refer to earlier screening data, capabilities, and programmes that may no longer be topical. We did not conduct a further systematic search for studies published after 2019 or systematic reviews published after March 2022. A systematic review published in July 2022 [69] also emphasised that there are currently no endorsed risk prediction models for breast cancer tailored to diverse ethnic populations.

Furthermore, we did not assess a machine learning-based software tool, the Mammo-Risk™ model (Predilife, Villejuif, France) [70], as it was published in 2022. The model was developed in the BCSC cohort [71, 72] to estimate the risk of developing breast cancer within the next five years based on the following four risk factors: age, family history of breast cancer, history of breast biopsies, and breast density with or without a polygenic risk score. Based on the results of the first validation studies, the model has an AUC value of 0.659 AUC and thus does not predict the individual risk of breast cancer with sufficient accuracy.

## 5. Conclusion

All breast cancer risk prediction models published to date show a limited ability to predict the individual breast cancer risk in women. Adding crucial risk factors, such as genetic information and breast density, only slightly improved the discrimination accuracy of the models. Hence, more reliable models with better predictive power are needed before using them in national screening programs. Besides, results of ongoing RCTs need to be awaited to shed more light on the benefit-harm ratio of risk-adjusted breast cancer screening compared to conventional age-based screening.

## Figures and Tables

**Table 1:** Criteria for a high risk of developing breast cancer.

Risk factor	Risk of developing breast cancer
First-degree relatives (e.g., parents and siblings) with a breast cancer diagnosis before the age of 50	Twofold risk
An increased breast density on mammography (D3—heterogenous density or D4—extreme density)	Women with extremely dense breast tissue have a twofold increased risk compared to women with an average density breast
Therapeutic thoracic radiation between the ages of 10 and 30	40% lifetime risk
History of atypical hyperplasia (AH)	The absolute cumulative risk of 30% at a 25-year follow-up
History of lobular in situ carcinoma (LCIS)	25% lifetime risk

Reference: table adapted based on [12].

**Table 2:** Inclusion and exclusion criteria following the PICO scheme.

	Inclusion	Exclusion
Population	Women between 25 and 75 years without suspected breast cancer	(i) Women with diagnosed breast cancer
		(ii) Men
Intervention	Predictive models to capture the individual breast cancer risk in a screening population	Models for predicting clinical outcomes of therapy or its response
Control	Prediction models compared with each other	—
Outcomes	Prognostic quality of the identified models:	
	(i) Discriminatory accuracy	
Study design	Systematic reviews (including systematic reviews with mixed ethnicities)	Systematic reviews that exclusively on a minority in Europe (e.g., Asian or African populations)
Publication date	Until March 2022	After March 2022
Language	English, German	Any other language

**Table 3:** Overview of the prognostic quality of the empirical and genetic models.

Risk prediction models and derived/modified versions	Number of validation studies	Discriminatory accuracy: AUC (reference), range^1^
*Empirical risk prediction models*		
Gail model/BRCAT	58	**0.41** [29]–**0.93** [30]
BCSC model	6	**0.58** [31]–**0.72** [32]
Rosner–Colditz model	9	**0.57** [33]–**0.68** [34]
*Genetic risk prediction models*		
IBIS/Tyrer–Cuzick model	8	**0.51** [35, 36]–**0.76** [37]
BOADICEA	1 [38]	**0.66** (without SNPs); **0.70** (SNP enhanced)
BRCAPRO™	1 [38]	**0.65** (without SNPs); **0.69** (SNP enhanced)

AUC = area under the curve, BCSC = Breast Cancer Surveillance Consortium, BRCAT = Breast Cancer Risk Assessment Tool, CI = confidence interval, IBIS = International Breast Cancer Intervention Study, NR = not reported, SNPs = single-nucleotide polymorphisms. ^1^Range involves AUC values for varying risk factors. The bold values present the AUC value ranges.

**Table 4:** Overview of the predictive quality of the original models.

Original models	Number of validation studies^1^	Discriminatory accuracy: AUC range^2^ (reference)
Barlow model	1 [27]	Premenopausal: **0.63**; postmenopausal: **0.62**
Models assessing the prognostic quality in invasive and/or in situ breast cancer	7^3^	**0.58** [39]–**0.71** [40]
Models assessing the prognostic quality in invasive and/or in situ breast cancer without and with breast density	5	Without breast density: **0.54** [41]–**0.65** [42, 43]; with breast density: **0.63** [41]–**0.72** [44]
Models assessing the prognostic quality in invasive and/or in situ breast cancer without and with SNPs	10	Without SNPs: **0.53** [45]–**0.79** [46]; SNPs enhanced: **0.60** [45]–**0.69** [47]
Model assessing the prognostic quality in ER-positive, HER2-negative, invasive, and noninvasive cancers without and with SNPs	1 [46]	Premenopausal: **0.708** (without SNPs) **0.785** (SNPs enhanced); postmenopausal: **0.693** (without SNPs) **0.764** (SNPs enhanced)

AUC = area under the curve, ER = oestrogen receptor, NR = not reported, SNPs = single-nucleotide polymorphisms. ^1^Studies assessing the prognostic quality in different populations considering various risk factors. ^2^Ranges involve AUC values for varying risk factors. ^3^Two studies reported no AUC values.

## Data Availability

The data used to support the findings of the overview of reviews are presented in the main text or in the Supplement.

## Supplementary Materials

Supplementary Materials Table S1: characteristics of the most important empirical and genetic breast cancer risk prediction models; Tables S2–S6: detailed search strategies; Table S7: characteristics of the included systematic reviews; Table S8: risk of bias assessment of the systematic reviews according to AMSTAR 2; Tables S9 a–f: extracted data on the primary study level for each breast cancer risk prediction model.

## References

[1] Bray F., Ferlay J., Soerjomataram I., Siegel R. L., Torre L. A., Jemal A. (2018). Global cancer statistics 2018: GLOBOCAN estimates of incidence and mortality worldwide for 36 cancers in 185 countries. *CA: A Cancer Journal for Clinicians*.

[2] Cancer Research Uk (2022). Breast cancer incidence by age. https://www.cancerresearchuk.org/health-professional/cancer-statistics/statistics-by-cancer-type/breast-cancer/incidence-invasive.

[3] Netherlands Cancer Registry (2022). Incidence of cancer in The Netherlands. http://www.dutchcancerfigures.nl/.

[4] Blows F. M., Driver K. E., Schmidt M. K. (2010). Subtyping of breast cancer by immunohistochemistry to investigate a relationship between subtype and short and long term survival: a collaborative analysis of data for 10,159 cases from 12 studies. *PLoS Medicine*.

[5] Waks A. G., Winer E. P. (2019). Breast cancer treatment: a review. *JAMA*.

[6] Nelson H. D., Zakher B., Cantor A. (2012). Risk factors for breast cancer for women aged 40 to 49 years: a systematic review and meta-analysis. *Annals of Internal Medicine*.

[7] Winters S., Martin C., Murphy D., Shokar N. K. (2017). Breast cancer epidemiology, prevention, and screening. *Prog Mol Biol Transl Sci*.

[8] Hartmann L. C., Sellers T. A., Frost M. H. (2005). Benign breast disease and the risk of breast cancer. *New England Journal of Medicine*.

[9] Collaborative Group on Hormonal Factors in Breast Cancer (1996). Breast cancer and hormonal contraceptives: collaborative reanalysis of individual data on 53,297 women with breast cancer and 100,239 women without breast cancer from 54 epidemiological studies. *Lancet*.

[10] (1997). Breast cancer and hormone replacement therapy: collaborative reanalysis of data from 51 epidemiological studies of 52,705 women with breast cancer and 108,411 women without breast cancer. Collaborative Group on Hormonal Factors in Breast Cancer. *Lancet*.

[11] Collaborative Group on Hormonal Factors in Breast Cancer (2001). Familial breast cancer: collaborative reanalysis of individual data from 52 epidemiological studies including 58,209 women with breast cancer and 101,986 women without the disease. *Lancet*.

[12] Marmot M. G., Altman D. G., Cameron D. A., Dewar J. A., Thompson S. G., Wilcox M. (2013). The benefits and harms of breast cancer screening: an independent review. *British Journal of Cancer*.

[13] (2016). Recommendations from European breast guidelines. https://ecibc.jrc.ec.europa.eu/recommendations/.

[14] Oeffinger K. C., Fontham E. T., Etzioni R. (2015). Breast cancer screening for women at average risk: 2015 guideline update from the American cancer society. *JAMA*.

[15] Myers E. R., Moorman P., Gierisch J. M. (2015). Benefits and harms of breast cancer screening: a systematic review. *JAMA*.

[16] Meads C., Ahmed I., Riley R. D. (2012). A systematic review of breast cancer incidence risk prediction models with meta-analysis of their performance. *Breast Cancer Research and Treatment*.

[17] Pavlou M., Ambler G., Seaman S. R. (2015). How to develop a more accurate risk prediction model when there are few events. *BMJ*.

[18] Ahmed I., Debray T. P., Moons K. G., Riley R. D. (2014). Developing and validating risk prediction models in an individual participant data meta-analysis. *BMC Medical Research Methodology*.

[19] Bleyer A., Welch H. G. (2012). Effect of three decades of screening mammography on breast-cancer incidence. *New England Journal of Medicine*.

[20] Paci E., Broeders M., Hofvind S., Puliti D., Duffy S. W. (2014). European breast cancer service screening outcomes: a first balance sheet of the benefits and harms. *Cancer Epidemiology, Biomarkers & Prevention*.

[21] Welch H. G., Passow H. J. (2014). Quantifying the benefits and harms of screening mammography. *JAMA Internal Medicine*.

[22] Canelo-Aybar C., Posso M., Montero N. (2022). Benefits and harms of annual, biennial, or triennial breast cancer mammography screening for women at average risk of breast cancer: a systematic review for the European Commission Initiative on Breast Cancer (ECIBC). *British Journal of Cancer*.

[23] Bond M., Pavey T., Welch K. (2013). Systematic review of the psychological consequences of false-positive screening mammograms. *Health Technology Assessment*.

[24] Amir E., Freedman O. C., Seruga B., Evans D. G. (2010). Assessing women at high risk of breast cancer: a review of risk assessment models. *JNCI Journal of the National Cancer Institute*.

[25] Quante A. S., Strahwald B., Fischer C., Kiechle M. (2018). Individualisiertes Brustkrebsrisiko – wie berechnen, wie bewerten und wie besprechen?. *Gynäkologe, Der*.

[26] Barlow W. E., White E., Ballard-Barbash R. (2006). Prospective breast cancer risk prediction model for women undergoing screening mammography. *Journal of the National Cancer Institute: Journal of the National Cancer Institute*.

[27] Bmj (2022). *Reporting Guideline for Overviews of Reviews of Healthcare Interventions: The Preferred Reporting Items for Overviews of Reviews (PRIOR) Statement*.

[28] Shea B. J., Reeves B. C., Wells G. (2017). Amstar 2: a critical appraisal tool for systematic reviews that include randomised or non-randomised studies of healthcare interventions, or both. *BMJ*.

[29] Stegeman I., Bossuyt P. M. (2012). Cancer risk models and preselection for screening. *Cancer Epidemiology*.

[30] Louro J., Posso M., Hilton Boon M. (2019). A systematic review and quality assessment of individualised breast cancer risk prediction models. *British Journal of Cancer*.

[31] Rockhill B., Byrne C., Rosner B., Louie M. M., Colditz G. (2003). Breast cancer risk prediction with a log-incidence model: evaluation of accuracy. *Journal of Clinical Epidemiology*.

[32] Vachon C. M., Pankratz V. S., Scott C. G. (2015). The contributions of breast density and common genetic variation to breast cancer risk. *Journal of the National Cancer Institute*.

[33] Rosner B., Colditz G. A., Iglehart J. D., Hankinson S. E. (2008). Risk prediction models with incomplete data with application to prediction of estrogen receptor-positive breast cancer: prospective data from the Nurses’ Health Study. *Breast Cancer Research*.

[34] Warwick J., Birke H., Stone J. (2014). Mammographic breast density refines Tyrer-Cuzick estimates of breast cancer risk in high-risk women: findings from the placebo arm of the International Breast Cancer Intervention Study I. *Breast Cancer Research*.

[35] Allman R., Dite G. S., Hopper J. L. (2015). SNPs and breast cancer risk prediction for African American and Hispanic women. *Breast Cancer Research and Treatment*.

[36] Amir E., Evans D. G., Shenton A. (2003). Evaluation of breast cancer risk assessment packages in the family history evaluation and screening programme. *Journal of Medical Genetics*.

[37] van Veen E. M., Brentnall A. R., Byers H. (2018). Use of single-nucleotide polymorphisms and mammographic density plus classic risk factors for breast cancer risk prediction. *JAMA Oncology*.

[38] Kaklamani V., Yi N., Sadim M. (2011). The role of the fat mass and obesity associated gene (FTO) in breast cancer risk. *BMC Medical Genetics*.

[39] Pruthi S., Heisey R. E., Bevers T. B. (2015). Chemoprevention for breast cancer. *Annals of Surgical Oncology*.

[40] Abdolell M., Tsuruda K. M., Lightfoot C. B., Payne J. I., Caines J. S., Iles S. E. (2016). Utility of relative and absolute measures of mammographic density vs clinical risk factors in evaluating breast cancer risk at time of screening mammography. *British Journal of Radiology*.

[41] Saikiran P., Ramzan R., Nandish S., Kamineni P. D., Priyanka, John A. M. (2019). Mammographic breast density assessed with fully automated method and its risk for breast cancer. *Journal of Clinical Imaging Science*.

[42] Bcrisktool (2022). *Gail Breast Cancer Risk Assessment Tool*.

[43] D’Orsi C. J. (2013). *ACR BI-RADS Atlas: Breast Imaging Reporting and Data System*.

[44] Sueta A., Ito H., Kawase T. (2012). A genetic risk predictor for breast cancer using a combination of low-penetrance polymorphisms in a Japanese population. *Breast Cancer Research and Treatment*.

[45] Guo J., Sueta A., Nakamura K. (2017). Genetic and environmental factors and serum hormones, and risk of estrogen receptor-positive breast cancer in pre- and postmenopausal Japanese women. *Oncotarget*.

[46] Eriksson M., Czene K., Pawitan Y., Leifland K., Darabi H., Hall P. (2017). A clinical model for identifying the short-term risk of breast cancer. *Breast Cancer Research*.

[47] Cintolo-Gonzalez J. A., Braun D., Blackford A. L. (2017). Breast cancer risk models: a comprehensive overview of existing models, validation, and clinical applications. *Breast Cancer Research and Treatment*.

[48] Anothaisintawee T., Teerawattananon Y., Wiratkapun C., Kasamesup V., Thakkinstian A. (2012). Risk prediction models of breast cancer: a systematic review of model performances. *Breast Cancer Research and Treatment*.

[49] Al-Ajmi K., Lophatananon A., Yuille M., Ollier W., Muir K. R. (2018). Review of non-clinical risk models to aid prevention of breast cancer. *Cancer Causes & Control*.

[50] Wang X., Huang Y., Li L., Dai H., Song F., Chen K. (2018). Assessment of performance of the Gail model for predicting breast cancer risk: a systematic review and meta-analysis with trial sequential analysis. *Breast Cancer Research*.

[51] Fung S. M., Wong X. Y., Lee S. X., Miao H., Hartman M., Wee H. L. (2019). Performance of single-nucleotide polymorphisms in breast cancer risk prediction models: a systematic review and meta-analysis. *Cancer Epidemiology, Biomarkers & Prevention*.

[52] Vilmun B. M., Vejborg I., Lynge E. (2020). Impact of adding breast density to breast cancer risk models: a systematic review. *European Journal of Radiology*.

[53] Gail M. H., Brinton L. A., Byar D. P. (1989). Projecting individualized probabilities of developing breast cancer for white females who are being examined annually. *JNCI Journal of the National Cancer Institute*.

[54] Vacek P. M., Skelly J. M., Geller B. M. (2011). Breast cancer risk assessment in women aged 70 and older. *Breast Cancer Research and Treatment*.

[55] Rong L., Li H., Wang E. L. (2016). To establish the breast cancer risk prediction model for women in Shenzhen in China Matern Child Health Care of China. *Maternal and child health in China*.

[56] Gail M. H., Costantino J. P., Pee D. (2007). Projecting individualized absolute invasive breast cancer risk in African American women. *JNCI Journal of the National Cancer Institute*.

[57] Keller B. M., Chen J., Daye D., Conant E. F., Kontos D. (2015). Preliminary evaluation of the publicly available Laboratory for Breast Radiodensity Assessment (LIBRA) software tool: comparison of fully automated area and volumetric density measures in a case-control study with digital mammography. *Breast Cancer Research*.

[58] Shieh Y., Hu D., Ma L. (2017). Joint relative risks for estrogen receptor-positive breast cancer from a clinical model, polygenic risk score, and sex hormones. *Breast Cancer Research and Treatment*.

[59] Shieh Y., Hu D., Ma L. (2016). Breast cancer risk prediction using a clinical risk model and polygenic risk score. *Breast Cancer Research and Treatment*.

[60] Zhang X., Rice M., Tworoger S. S. (2018). Addition of a polygenic risk score, mammographic density, and endogenous hormones to existing breast cancer risk prediction models: a nested case-control study. *PLoS Medicine*.

[61] Dite G. S., MacInnis R. J., Bickerstaffe A. (2016). Breast cancer risk prediction using clinical models and 77 independent risk-associated SNPs for women aged under 50 years: Australian breast cancer family registry. *Cancer Epidemiology, Biomarkers & Prevention*.

[62] Wang J., Azziz A., Fan B. (2013). Agreement of mammographic measures of volumetric breast density to MRI. *PLoS One*.

[63] Gubern-Mérida A., Kallenberg M., Platel B., Mann R. M., Martí R., Karssemeijer N. (2014). Volumetric breast density estimation from full-field digital mammograms: a validation study. *PLoS One*.

[64] Alonzo-Proulx O., Mawdsley G. E., Patrie J. T., Yaffe M. J., Harvey J. A. (2015). Reliability of automated breast density measurements. *Radiology*.

[65] Brinton J. T., Hendrick R. E., Ringham B. M., Kriege M., Glueck D. H. (2019). Improving the diagnostic accuracy of a stratified screening strategy by identifying the optimal risk cutoff. *Cancer Causes & Control*.

[66] Gail M. H., Pfeiffer R. M. (2005). On criteria for evaluating models of absolute risk. *Biostatistics*.

[67] Esserman L. J., Anton-Culver H., Borowsky A. (2017). The WISDOM Study: breaking the deadlock in the breast cancer screening debate. *NPJ Breast Cancer*.

[68] Horizon (2022). International randomized study comparing personalized, risk-stratified to standard breast cancer screening in women aged 40–70. https://cordis.europa.eu/project/rcn/212694/factsheet/en.

[69] Zheng Y., Li J., Wu Z. (2022). Risk prediction models for breast cancer: a systematic review. *BMJ Open*.

[70] Saghatchian M., Abehsera M., Yamgnane A. (2022). Feasibility of personalized screening and prevention recommendations in the general population through breast cancer risk assessment: results from a dedicated risk clinic. *Breast Cancer Research and Treatment*.

[71] Dartois L., Gauthier É, Heitzmann J. (2015). A comparison between different prediction models for invasive breast cancer occurrence in the French E3N cohort. *Breast Cancer Research and Treatment*.

[72] Gauthier E., Tice J. A., Michiels S., Kaufmanis A., Drubay D., Brixi Z. (2017). Breast cancer risk prediction by a machine learning model versus the BCSC score: performances on the US Breast Cancer Screening Consortium and a French screening cohort. *Cancer Research*.

[73] Frühwirth I., Wolf S. (2022). *Risikobasiertes Brustkrebs-Screening in Österreich: Systematische Analyse der Vorhersagemodelle zur Erfassung des individuellen Brustkrebsrisikos, deren Nutzen und Anwendbarkeit im Brustkrebs-Screening Programm*.

[74] Gms (2023). *24 Jahrestagung des Netzwerks Evidenzbasierte Medizin e V*.

