# Supplementary material for: The Prognostic Quality of Risk Prediction Models to Assess the Individual Breast Cancer Risk in Women: An Overview of Reviews
Source: Breast J. 2024 Mar 21;2024:1711696. doi: 10.1155/2024/1711696 (PMC10978083; doi:10.1155/2024/1711696)
Supplement: Supplementary Materials — Table S1: characteristics of the most important empirical and genetic breast cancer risk prediction models; Tables S2–S6: detailed search strategies; Table S7: characteristics of the included systematic reviews; Table S8: risk of bias assessment of the systematic reviews according to AMSTAR 2; Tables S9 a–f: extracted data on the primary study level for each breast cancer risk prediction model. [file 1711696.f1.docx]

**Table S1**: Characteristics of the most important breast cancer risk prediction models

| **a)** | **Empirical prediction models** | | | | | | |  |
| --- | --- | --- | --- | --- | --- | --- | --- | --- |
|  | **Gail/BRCAT/NCI [2]** | **Barlow/BCSC pre-menopausal [3]** | **Barlow/BCSC post-menopausal [3]** | **BCSC/Tice  [4, 5]** | | **Rosner-Colditz  [6-9]** | |  |
| **Included risk factors** | | | | | | | |  |
| *Personal information* | | | | | | | |  |
| Age | √(>35years) | √ | √ | √ (>35years) | | √ | |  |
| Body Mass Index/weight |  |  | √ |  | | √ | |  |
| Alcohol consumption |  |  |  |  | |  | |  |
| *Hormonal/reproductive factors* |  |  |  |  | |  | |  |
| Age at menarche | √ |  |  |  | |  | |  |
| Age of 1^st^ life birth | √ |  |  |  | |  | |  |
| Age of menopause |  |  | √ |  | |  | |  |
| Parity |  |  |  |  | |  | |  |
| Hormone replacement therapy |  |  | √ |  | | √ | |  |
| Oral contraceptive use |  |  |  |  | |  | |  |
| *Personal breast disease* | | | | | | | |  |
| Breast density | Included in Chen model [10] | √ | √ | √ | |  | |  |
| Atypical ductal hyperplasia | √ |  |  |  | |  | |  |
| Lobular carcinoma in situ |  |  |  |  | |  | |  |
| Prior breast biopsies | √ | √ | √ | √ | |  | |  |
| Benign breast disease |  |  |  | √ | | √ | |  |
| *Family history* | | | | | | | |  |
| Degree relatives assessed | 1^st^ | 1^st^ | 1^st^ | 1^st^ | | 1^st^ | |  |
| Age at breast cancer diagnosis in a relative |  |  |  |  | |  | |  |
| Bilateral breast cancer |  |  |  |  | |  | |  |
| Male breast cancer |  |  |  |  | |  | |  |
| Ovarian cancer |  |  |  |  | |  | |  |
| *Race/ethnicity* | √ |  | √ | √ | |  | |  |
| *Genetic testing* | | | | | | | |  |
| BRCA1/2 |  |  |  |  | |  | |  |
| SNPs | has been modelled |  |  | has been modelled | |  | |  |
| **Further characteristics** | | | | | | | |  |
| *Prediction horizon* | 5-year lifetime (up to age 90) |  |  | 5-year 10-year | |  | |  |
| *Web-based tool/domain* | <https://bcrisktool.cancer.gov/calculator.html>  freely accessible | not available as a web-based tool | | [https://tools.bcsc-scc.org/ BC5yearRisk/calculator.htm](https://tools.bcsc-scc.org/BC5yearRisk/calculator.htm)  freely accessible for healthcare professionals | | not available via a specific software, can be calculated using standard statistical packages | |  |
| *Language* | english |  | | english | |  | |  |
| *Not applicable for* | - <35 or >74 - women with BRCA1 or BRCA2 mutation - previous history of DCIS breast cancer - women with a history  of LCIS | previous diagnosis of breast cancer or DCIS or prior breast augmentation or mastectomy | | - <35 or >74 - previous diagnosis of breast cancer of DCIS - previous breast augmentation - previous mastectomy | |  | |  |
| *Best use* | to determine if a patient is a candidate for risk-reducing medications, e.g. tamoxifen or raloxifene [11] |  | | breast cancer risk prediction for most patients except for those with significant family history [11] | |  | |  |
| **b)** | **Genetic prediction model** | | | | | | | |
|  | BOADICEA [12, 13] | BRCAPRO™ [14] | | | IBIS/Tyrer-Cuzick [15, 16] | | Claus [17] | |
| **Included risk factors** | | | | | | | | |
| *Personal information* | | | | | | | | |
| Age | √ | √ | | | √ | | √ | |
| Body Mass Index/weight | included v6 |  | | | √ | |  | |
| Alcohol consumption | included v6 |  | | |  | |  | |
| *Hormonal/reproductive factors* | | | | | | | | |
| Age at menarche | included v6 |  | | | √ | |  | |
| Age of 1^st^ life birth | included v6 |  | | | √ | |  | |
| Age of menopause | included v6 |  | | | √ | |  | |
| Parity | included v6 |  | | | √ | |  | |
| Hormone replacement therapy | included v6 |  | | | √ | |  | |
| Oral contraceptive use | included v6 |  | | |  | |  | |
| *Personal breast disease* | | | | | | | | |
| Breast density | included v6 |  | | | √ | |  | |
| Atypical ductal hyperplasia |  |  | | | √ | |  | |
| Lobular carcinoma in situ |  |  | | | √ | |  | |
| Prior breast biopsies |  |  | | | √ | |  | |
| Benign breast disease |  |  | | |  | |  | |
| *Family history* | | | | | | | | |
| Degree relatives assessed* | any | any | | | 1^st^, 2^nd^ | | 1^st^, 2^nd^ | |
| Age at breast cancer diagnosis in a relative | √ | √ | | | √ | | √ | |
| Bilateral breast cancer |  | √ | | | √ | |  | |
| Male breast cancer | √ | √ | | |  | |  | |
| Ovarian cancer | √ | √ | | | √ | |  | |
| *Race/ethnicity* |  | √ | | |  | |  | |
| *Genetic testing* | | | | | | | | |
| BRCA1/2 | √ | √ | | | √ | |  | |
| SNPs | has been modelled | has been modelled | | | has been modelled | |  | |
| Further characteristics | | | | | | | | |
| *Prediction horizon* | 5-year  10-year  lifetime (up to age 80) |  | | | 10-year  lifetime (up to age 85) | |  | |
| *Web-based tool/domain* | https://ccge.medschl.cam.ac.uk/ boadicea/ research tool and not licensed to support clinical or diagnostic decisions  open access, registration required: <https://www.canrisk.org/de/> | software is free for non-clinical research using the R package; for commercial use license is required;  a license is required for commercial use: https://projects.iq.harvard.edu/bayesmendel/BRCAPRO™ | | | for research purposes for invasive breast cancer or DCIS: <http://www.ems-trials.org/riskevaluator/>  for clinical use: <https://ibis-risk-calculator.magview.com/>  https://ibis.ikonopedia.com | | not available | |
| *Language* | English, German | English | | | English | |  | |
| *Not applicable for* |  | women with average breast cancer risk without a family history | | | women who have already been diagnosed with breast cancer or DCIS | |  | |
| *Best use* | a comprehensive model for calculating the future risk of breast or ovarian cancer and mutations in moderate- or high-risk genes | assessing the probability of a deleterious germline mutation of the BRCA1/2 genes, decision aid whether to undergo genetic testing | | | decision-making about genetic counselling, breast cancer risk prediction for patients with a family history of young age (<50) at diagnosis of cancer, second-degree relatives with breast or ovarian cancers, male breast cancers, and ovarian cancer [11] | |  | |

*Abbreviations: BCSC=Breast Cancer Surveillance Consortium, BOADICEA=Breast and Ovarian Analysis of Disease Incidence and Carrier Estimation Algorithm, BRCAT=Breast Cancer Risk Assessment Tool, DCIS=ductal carcinoma in situ, IBIS=International Breast Cancer Intervention Study, LCIS=lobular in situ carcinoma, NCI=National Cancer Institute, SNPs=single nucleotide polymorphisms, v6=version 6*

**Table S2**: Search strategy Ovid MEDLINE

| Database: Ovid MEDLINE(R) and In-Process, In-Data-Review & Other Non-Indexed Citations and Daily <1946 to March 15, 2022>,  Ovid MEDLINE(R) and Epub Ahead of Print, In-Process, In-Data-Review & Other Non-Indexed Citations and Daily <2018 to March 15, 2022> | |
| --- | --- |
| Search date: 16.03.2022 | |
| ID | Search |
| 1 | exp Breast Neoplasms/(378317) |
| 2 | (breast* adj2 (cancer* or carcinoma* or tumo?r* or neoplasm* or malig* or metasta*)).mp. (535317) |
| 3 | 1 or 2 (535523) |
| 4 | exp Mass Screening/(160714) |
| 5 | screening*.mp. (907086) |
| 6 | exp Mammography/(36172) |
| 7 | mammograph*.mp. (49803) |
| 8 | 4 or 5 or 6 or 7 (946354) |
| 9 | 3 and 8 (66871) |
| 10 | ((cancer* or carcinoma* or tumo?r* or neoplasm* or malig* or metasta*) adj4 risk*).mp. (252692) |
| 11 | 9 and 10 (10029) |
| 12 | (risk* adj3 (predict* or forecast* or estimat* or model*)).mp. (189244) |
| 13 | *Models, Theoretical/(72049) |
| 14 | (predict* or forecast* or estimat*).mp. (4124989) |
| 15 | 13 and 14 (26655) |
| 16 | 12 or 15 (214583) |
| 17 | 11 and 16 (1485) |
| 18 | limit 17 to (english or german) (1466) |
| 19 | limit 17 to yr=„2012 - 2022“ (1089) |
| 20 | remove duplicates from 19 (695) |
| 21 | limit 20 to (comment or editorial or letter) (6) |
| 22 | 20 not 21 (689) |

**Table S3**: Search strategy Cochrane

| Search Name: Brustkrebsscreening Vorhersagemodelle | |
| --- | --- |
| Last saved: 17/03/2022 16:37:01 | |
| Comment: ISF 170322 | |
| ID | Search |
| #1 | MeSH descriptor: [Breast Neoplasms] explode all trees |
| #2 | (breast* NEAR (cancer* OR carcinoma* OR tumor* OR tumour* OR neoplasm* OR malig* OR metasta*)):ti,ab,kw (Word variations have been searched) |
| #3 | #1 OR #2 |
| #4 | MeSH descriptor: [Mass Screening] explode all trees |
| #5 | (screening*):ti,ab,kw (Word variations have been searched) |
| #6 | MeSH descriptor: [Mammography] explode all trees |
| #7 | (mammograph*):ti,ab,kw (Word variations have been searched) |
| #8 | #4 OR #5 OR #6 OR #7 |
| #9 | #3 AND #8 |
| #10 | ((cancer* OR carcinoma* OR tumor* OR tumour* OR neoplasm* OR malig* OR metasta*) NEAR risk*):ti,ab,kw (Word variations have been searched) |
| #11 | #9 AND #10 |
| #12 | (risk* NEAR (predict* OR forecast* OR estimat* OR model*)):ti,ab,kw (Word variations have been searched) |
| #13 | MeSH descriptor: [Models, Theoretical] explode all trees |
| #14 | (predict* OR forecast* OR estimat*):ti,ab,kw (Word variations have been searched) |
| #15 | #13 AND #14 |
| #16 | #12 OR #15 |
| #17 | #11 AND #16 with Cochrane Library publication date Between Jan 2012 and Mar 2022 |
| #18 | #11 AND #16 with Publication Year from 2012 to 2022, in Trials |
| #19 | #17 OR #18 |
| #20 | (conference abstract):pt (Word variations have been searched) |
| #21 | (abstract):so (Word variations have been searched) |
| #22 | (clinicaltrials OR trialsearch OR ANZCTR OR ensaiosclinicos OR Actrn OR chictr OR cris OR ctri OR registroclinico OR clinicaltrialsregister OR DRKS OR IRCT OR Isrctn OR rctportal OR JapicCTI OR JMACCT OR jRCT OR JPRN OR Nct OR UMIN OR trialregister OR PACTR OR R.B.R.OR REPEC OR SLCTR OR Tcr):so (Word variations have been searched) |
| #23 | #20 OR #21 OR #22 |
| #24 | #19 NOT #23 |
| Total hits: 58 | |

**Table S4**: Search strategy CRD

| Search Name: Brustkrebsscreening_Vorhersagemodelle | |
| --- | --- |
| Search date: 17.03.2022 | |
| ID | Search |
| 1 | MeSH DESCRIPTOR Breast Neoplasms EXPLODE ALL TREES |
| 2 | (breast* NEAR (cancer* OR carcinoma* OR tumor* OR tumour* OR neoplasm* OR malig* OR metasta*)) |
| 3 | #1 OR #2 |
| 4 | MeSH DESCRIPTOR Mass Screening EXPLODE ALL TREES |
| 5 | (screening*) |
| 6 | MeSH DESCRIPTOR Mammography EXPLODE ALL TREES |
| 7 | (mammograph*) |
| 8 | #4 OR #5 OR #6 OR #7 |
| 9 | #3 AND #8 |
| 10 | ((cancer* OR carcinoma* OR tumor* OR tumour* OR neoplasm* OR malig* OR metasta*) NEAR risk*) |
| 11 | #9 AND #10 |
| 12 | (risk* NEAR (predict* OR forecast* OR estimat* OR model*)) |
| 13 | MeSH DESCRIPTOR Models, Theoretical EXPLODE ALL TREES |
| 14 | (predict* OR forecast* OR estimat*) |
| 15 | #13 AND #14 |
| 16 | #12 OR #15 |
| 17 | #11 AND #16 |
| 18 | (#17) FROM 2012 TO 2022 |
| Total hits: 4 | |

**Table S5**: Search strategy HTA-INAHTA

| Date of search: 17.03.2022 | |
| --- | --- |
| ID | Search step #,Search query,“Hits“,“Searched At“ |
| 21 | ((English)[Language] OR (German)[Language]) AND ((* FROM 2012 TO 2022) AND ((((predict* OR forecast* OR estimat*) AND („Models Theoretical“[mhe])) OR ((risk*) AND (predict* OR forecast* OR estimat* OR model*))) AND (((cancer* OR carcinoma* OR tumor* OR tumour* OR neoplasm* OR malig* OR metasta*) AND (risk*)) AND (((mammograph*) OR („Mammography“[mhe]) OR (screening*) OR („Mass Screening“[mhe])) AND (((breast*) AND (cancer* OR carcinoma* OR tumor* OR tumour* OR neoplasm* OR malig* OR metasta*)) OR („Breast Neoplasms“[mhe])))))),“7“,“2022-03-17T16:28:16.000000Z“ |
| 20 | (English)[Language] OR (German)[Language],“15218“,“2022-03-17T16:28:02.000000Z“ |
| 19 | (* FROM 2012 TO 2022) AND ((((predict* OR forecast* OR estimat*) AND („Models Theoretical“[mhe])) OR ((risk*) AND (predict* OR forecast* OR estimat* OR model*))) AND (((cancer* OR carcinoma* OR tumor* OR tumour* OR neoplasm* OR malig* OR metasta*) AND (risk*)) AND (((mammograph*) OR („Mammography“[mhe]) OR (screening*) OR („Mass Screening“[mhe])) AND (((breast*) AND (cancer* OR carcinoma* OR tumor* OR tumour* OR neoplasm* OR malig* OR metasta*)) OR („Breast Neoplasms“[mhe]))))),“11“,“2022-03-17T16:26:34.000000Z“ |
| 18 | * FROM 2012 TO 2022,“7683“,“2022-03-17T16:26:00.000000Z“ |
| 17 | (((predict* OR forecast* OR estimat*) AND („Models Theoretical“[mhe])) OR ((risk*) AND (predict* OR forecast* OR estimat* OR model*))) AND (((cancer* OR carcinoma* OR tumor* OR tumour* OR neoplasm* OR malig* OR metasta*) AND (risk*)) AND (((mammograph*) OR („Mammography“[mhe]) OR (screening*) OR („Mass Screening“[mhe])) AND (((breast*) AND (cancer* OR carcinoma* OR tumor* OR tumour* OR neoplasm* OR malig* OR metasta*)) OR („Breast Neoplasms“[mhe])))),“25“,“2022-03-17T16:24:53.000000Z“ |
| 16 | ((predict* OR forecast* OR estimat*) AND („Models Theoretical“[mhe])) OR ((risk*) AND (predict* OR forecast* OR estimat* OR model*)),“937“,“2022-03-17T16:24:27.000000Z“ |
| 15 | (predict* OR forecast* OR estimat*) AND („Models Theoretical“[mhe]),“20“,“2022-03-17T16:24:14.000000Z“ |
| 14 | predict* OR forecast* OR estimat*,“1824“,“2022-03-17T16:24:06.000000Z“ |
| 13 | „Models Theoretical“[mhe],“126“,“2022-03-17T16:21:50.000000Z“ |
| 12 | (risk*) AND (predict* OR forecast* OR estimat* OR model*),“926“,“2022-03-17T16:20:31.000000Z“ |
| 11 | ((cancer* OR carcinoma* OR tumor* OR tumour* OR neoplasm* OR malig* OR metasta*) AND (risk*)) AND (((mammograph*) OR („Mammography“[mhe]) OR (screening*) OR („Mass Screening“[mhe])) AND (((breast*) AND (cancer* OR carcinoma* OR tumor* OR tumour* OR neoplasm* OR malig* OR metasta*)) OR („Breast Neoplasms“[mhe]))),“74“,“2022-03-17T16:19:59.000000Z“ |
| 10 | (cancer* OR carcinoma* OR tumor* OR tumour* OR neoplasm* OR malig* OR metasta*) AND (risk*),“700“,“2022-03-17T16:19:48.000000Z“ |
| 9 | ((mammograph*) OR („Mammography“[mhe]) OR (screening*) OR („Mass Screening“[mhe])) AND (((breast*) AND (cancer* OR carcinoma* OR tumor* OR tumour* OR neoplasm* OR malig* OR metasta*)) OR („Breast Neoplasms“[mhe])),“191“,“2022-03-17T16:19:10.000000Z“ |
| 8 | (mammograph*) OR („Mammography“[mhe]) OR (screening*) OR („Mass Screening“[mhe]),“1387“,“2022-03-17T16:18:51.000000Z“ |
| 7 | mammograph*,“139“,“2022-03-17T16:18:34.000000Z“ |
| 6 | „Mammography“[mhe],“113“,“2022-03-17T16:18:17.000000Z“ |
| 5 | screening*,“1256“,“2022-03-17T16:17:53.000000Z“ |
| 4 | „Mass Screening“[mhe],“768“,“2022-03-17T16:17:31.000000Z“ |
| 3 | ((breast*) AND (cancer* OR carcinoma* OR tumor* OR tumour* OR neoplasm* OR malig* OR metasta*)) OR („Breast Neoplasms“[mhe]),“763“,“2022-03-17T16:16:48.000000Z“ |
| 2 | (breast*) AND (cancer* OR carcinoma* OR tumor* OR tumour* OR neoplasm* OR malig* OR metasta*),“706“,“2022-03-17T16:16:36.000000Z“ |
| 1 | „Breast Neoplasms“[mhe],“545“,“2022-03-17T16:14:59.000000Z“ |
| Total hits: 7 | |

**Table S6**: Search strategy Embase

| Search date: 16 Mar 2022 | | |
| --- | --- | --- |
| No. | Query Results | Results |
| #23. | #21 NOT #22 | 421 |
| #22. | #21 AND (‘Conference Abstract’/it OR ‘Editorial’/it OR ‘Letter’/it OR ‘Note’/it) | 194 |
| #21. | #20 AND ([[8](#_ENREF_8)]/lim OR [german]/lim) AND [2012-2022]/py | 615 |
| #20. | #19 AND ([[8](#_ENREF_8)]/lim OR [german]/lim) | 813 |
| #19. | #17 OR #18 | 816 |
| #18. | (‘risk* prediction model*’ NEAR/2 (‘breast* cancer*’ OR ‘breast* carcinoma*’ OR ‘breast* tumo*r*’ OR ‘breast* neoplasm*’ OR ‘breast* malig*’ OR ‘breast* metasta*’)):ti,ab | 141 |
| #17. | #11 AND #16 | 747 |
| #16. | #12 OR #13 OR #14 OR #15 | 74,224 |
| #15. | (risk* NEAR/1 (predict* OR forecast* OR estimat* OR model*)):ti,ab | 74,104 |
| #14. | ‘prediction model’/exp | 123 |
| #13. | ‘risk prediction model’/exp | 56 |
| #12. | ‘risk prediction’/exp | 28 |
| #11. | #9 AND #10 | 5,928 |
| #10. | ((cancer* OR carcinoma* OR tumo*r* OR neoplasm* OR malig* OR metasta*) NEAR/1 risk*):ti,ab | 84,199 |
| #9. | #3 AND #8 | 84,997 |
| #8. | #4 OR #5 OR #6 OR #7 | 1,249,415 |
| #7. | mammograph*:ti,ab | 40,551 |
| #6. | ‘mammography’/exp | 62,836 |
| #5. | screening*:ti,ab | 848,123 |
| #4. | ‘screening’/exp | 758,789 |
| #3. | #1 OR #2 | 630,359 |
| #2. | (breast* NEAR/1 (cancer* OR carcinoma* OR tumo*r* OR neoplasm* OR malig* OR metasta*)):ti,ab | 485,046 |
| #1. | ‘breast cancer’/exp | 526,598 |

**Table S7**: Characteristics of the included systematic reviews

| Author year  [reference] | Anothaisintawee  2012 [18] | Meads  2012 [19] | Stegeman  2012 [20] | Al-Ajmi  2018 [21] | Wang  2018 [22] | Louro  2019 [23] | Fung  2019 [24] | Vilmun  2020 [25] |
| --- | --- | --- | --- | --- | --- | --- | --- | --- |
| Country of first author's affiliation | Thailand | UK | Netherlands | UK | China | Spain | Singapore | Denmark |
| Conflicts of  interest/ funding | Yes/supported by the Health Intervention and Technology Assessment Program, the Thai Health Promotion Foundation, the Health Systems Research Institute, the Bureau of Policy and Strategy of the Ministry of Public Health, and the Thai Health-Global Link Initiative Project | None/NR | None/NR | None/Integrative Cancer Epidemiology Programme | None/7 Chinese grants, in part by the Program for Changjiang Scholars and Innovative Research Team at University in China | None/partially supported by Agència de Qualitat i Avaluació Sanitàries de Catalunya (AQuAS) and by grants from Instituto de Salud Carlos III FEDER | None/Singapore Ministry of Health Services Research Competitive Research Grant, administered by the National Medical Research Council | NR/Eurostars, E9714 IBSCREEN |
| Aim | SR of the development and performance of globally existing prediction models, which are used to estimate the risk of breast cancer. | Qualitative summary of models and the risk factors they contain, and a meta-analysis of model performance statistics across studies, to allow the performance of each model to be compared. | Examination of the validity and performance of cancer risk models and whether they have been used and evaluated in defining inclusion criteria for risk-based cancer screening programs. | Focus on breast cancer risk prediction models that incorporate modifiable risk factors and/or factors that can be self-reported. | Evaluation of the performance of different versions of the Gail model by means of SR and MA. | SR and quality assessment of models addressed to women in the general population. | Identification of existing SNP-enhanced breast cancer risk prediction models, assess their performance, and evaluate the extent of improvement in performance, from the addition of genetic information in the SNP-enhanced risk models. | Identification of clinical breast cancer risk prediction models reporting outcomes with and without the inclusion of mammographic density. |
| Inclusion criteria | >1 risk factor, the outcome as breast cancer versus non-breast cancer, applied any regression equation, reported each model's performance | Any studies developing and/or validating a breast cancer risk prediction model for the general female population using multiple variables, at least one of which was a modifiable risk factor | Multivariable risk models for breast cancer, any study, variables without additional testing (information obtained via questionnaires), development or evaluation of the model based on empirical data collected in a series of screenees or study participants | Only risk models with non-clinical factors (modifiable risk factors), used by women themselves | Studies 1) validating the performance of the original or modified Gail model, 2) providing the E/O ratio (95% CI) or sufficient data for calculating E/O ratio focusing on cohort studies, 3) providing the AUC (95% CI), 4) sample size >100, 5) mean follow-up for cohort studies > 1yr, when multiple publications included the same population, larger sample size or longer  follow-up periods were included, 6) MA including publications provided sufficient data for calculating TP, FP, FN, TN values | Models assessing >1 risk factor reported the quantitative characteristics of the risk prediction model; if multiple publications based on the same risk model, the most extensive report of the model in terms of risk factors reported were chosen | Studies 1) published in English, 2) assessing risk prediction models for breast cancer in women, which reported outcomes using AUC or its equivalent – C-statistic and/or NRI | Studies 1) developed or modified existing clinical breast cancer risk prediction models applicable to the general population of women, 2) to have the same risk model with breast density included as an additional risk factor for comparison, 3) no restriction on study type, 4) published in English |
| Exclusion criteria | NR | Breast cancer in men, women who already had breast cancer or benign breast pathology when recruited; studies in high-risk groups of women, e.g. with specific genetic mutations or who have close family relatives with breast cancer, models published before 1985 | Models containing laboratory measurements | Clinical (any variable which needs physician input, e.g., presence of atypical hyperplasia) and any genetic risk factors | NR | External validation studies that replicated previous models without adding any additional information | Genome-wide association studies, reviews, narratives, prognostic or diagnostic studies, model development studies, non-risk prediction studies and studies that included only BRCA mutation carriers | NR |
| Literature search in the following databases | Medline, EMBASE | Cochrane library, MEDLINE, EMBASE, CAB Abstracts and PsychINFO | Medline, EMBASE | PubMed, ScienceDirect, the Cochrane Database of Systematic Reviews | PubMed, EMBASE, WANFANG [26],  VIP [27], China National Knowledge Infrastructure (CNKI) [28] | Medline, the Cochrane Library, EMBASE | EMBASE, Scopus, PubMed | PubMed, EMBASE, Web of  Science, the Cochrane Library |
| Included study designs (number) | Cohort (10), case-control (6), nested case-control (2) | NR | Cohort (3), case-control (7), nested case-control (1), observational study (1) | Case-control (9), cohort (3), longitudinal (1), prospective (1) | Calibration of Gail model: NR;  Discrimination of Gail model: cohort (21), case-control (5), cross-sectional (3);  Diagnostic accuracy of the Gail model: cohort (4), case-control (7), cross-sectional (1), nested case-control (1) | Case-control (13), prospective cohorts (5), retrospective cohorts (4), risk estimates obtained from a SR of the literature (2) | Case-control (26) | Cohort (4), case-control (8) |
| Quality assessment/ tool to assess risk of bias or quality | NR | Quality assessment/Altman [29] | NR | Degree of confidence in variables/Harvard report [8] | Newcastle–Ottawa Scale (NOS) [30],  Quality Assessment Diagnostic Accuracy Studies (QUADAS) [31] | Risk of bias and quality assessment/ ISPOR-AMCP-NPC Questionnaire [32] | 25-item checklist "Strengthening the Reporting of Genetic RIsk Prediction Studies "(GRIPS) statement [33], Newcastle-Ottawa Quality Assessment Scale (NOS) [34] | NR |
| Total number of included studies/ models (published between) | 25 (reported only 18 in detail)/7 (1989-2010) | 26/17 (1989-2010) | 12/4 (1989-2007) | 14/3 (1989-2013) | 24 for calibration of Gail model (1994-2015) 26 for discrimination of Gail model (2001-2016) 13 for diagnostic accuracy of the Gail model (2001-2016) | 24/8 (1989-2018) | 26 (25 included in MA)/ 17 (2010-2018) | 12/7 (2011-2018) |
| Number of included studies/ population (study size) | 9/Caucasian (381,144), 2/Asian (1,356), 1/African–Americans (3,281), 6/mixed populations (3,293,062) | NR | 8/White (241,995),  3/African-Americans (5,587),  1/mixed populations (3,236), 2/NR (2,480) | 7/Caucasian (417,270), 5/Asian (15,153), 1/African (3,254), 1/Hispanic (135,329) | NR | 11/Caucasian (328,826), 1/European (4,598), 2/Asian (2,347), 1/Asian (NR), 1/African-American (3,254), 1/Hispanic (2,497), 7/multi ethnicities (5,492,893), 1/multi ethnicities (NR) | 13/Caucasian or European (93,874), 8/Asian (61,562), 3/mixed populations (1,973), 1/Australians (1,425), 1/Afican-American and Hispanics (10,769) | 4/European (62,210), 4/American (134,872), 2/Asian (24,811), 2/NR (2,448) |
| Evaluation measures | Calibration (E/O ratio along with 95% CI, or goodness of fit test), discrimination (ROC analysis, C-statistic along with 95% CI) | Calibration, C-statistic (discrimination performance), MA of E/O ratio, MA of the C-statistic | Discrimination, calibration, accuracy, distribution of risk | Calibration, discriminatory accuracy, utility | Discrimination, calibration, diagnostic accuracy | Calibration, discrimination | Discriminatory accuracy (AUC), improvement in predictive ability (NRI) | Discrimination, calibration without and with mammographic density |
| Range of risk factors  of included models | 4-13 | 4-13 | 4-10 | 5-16 | NR | 5-18 | 2-10 | 4-10 |
| Remarks |  |  | Evaluated multivariable risk models for breast cancer, but also for cervical cancer and colon cancer. |  |  |  |  |  |

*Abbreviations: AUC=area under the curve, FN=false-negative, FP=false-positive, MA=meta-analysis, NR=not reported, ROC=receiver operating characteristic curve, SR=systematic revview, TN=true-negative, TP=true-positive, UK=United Kingdom*

**Table S8**: Quality assessment of the systematic review according to AMSTAR 2

| **Quality Assessment Check AMSTAR 2** | **Author year [reference]** | | | | | | | |
| --- | --- | --- | --- | --- | --- | --- | --- | --- |
|  | **Anothaisintawee 2012 [18]** | **Meads  2012 [19]** | **Stegemann 2012 [20]** | **Al-Ajmi  2018 [21]** | Wang  2018 [22] | Louro  2019 [23] | Fung  2019 [24] | Vilmun  2020 [25] |
| Did the research questions and inclusion criteria for the review include the components of PICO? | No | No | No | No | No | Yes | No | Yes |
| Did the report of the review contain an explicit statement that the review methods were established prior to the conduct of the review and did the report justify any significant deviations from the protocol? | No | Yes | No | No | Yes | Yes | No | Partial yes |
| Did the review authors explain their selection of the study designs for inclusion in the review? | No | Yes | Yes | No | Yes | Yes | Yes | Yes |
| Did the review authors use a comprehensive literature search strategy? | No | Yes | Partial yes | Yes | Yes | Yes | Partial yes | Yes |
| Did the review authors perform study selection in duplicate? | No | Yes | Yes | No | Yes | Yes | Yes | Yes |
| Did the review authors perform data extraction in duplicate? | No | Yes | No | No | Yes | Yes | No | Yes |
| Did the review authors provide a list of excluded studies and justify the exclusions? | No | No | No | Partial yes | Partial yes | Yes | Partial yes | Partial yes |
| Did the review authors describe the included studies in adequate detail? | Yes | Yes | Yes | Yes | Yes | Yes | Yes | Yes |
| Did the review authors use a satisfactory technique for assessing the risk of bias in individual studies that were included in the review? | No | No | No | No | Partial yes | Yes | Yes | No |
| Did the review authors report on the sources of funding for the studies included in the review? | No | No | No | No | No | Yes | No | No |
| If meta-analysis was performed did the review authors use appropriate methods for statistical combination of results? | NA | Yes | NA | NA | Yes | NA | Yes | NA |
| If meta-analysis was performed, did the review authors assess the potential impact of risk of bias in individual studies on the results of the meta-analysis or other evidence synthesis? | NA | No | NA | NA | No | NA | No | NA |
| Did the review authors account for risk of bias in individual studies when interpreting/discussing the results of the review? | NA | No | NA | NA | No | NA | Yes | NA |
| Did the review authors provide a satisfactory explanation for, and discussion of, any heterogeneity observed in the results of the review? | NA | Yes | NA | NA | Yes | NA | Yes | NA |
| If they performed quantitative synthesis did the review authors carry out an adequate investigation of publication bias (small study bias) and discuss its likely impact on the results of the review? | NA | No | NA | NA | Yes | NA | No | NA |
| Did the review authors report any potential sources of conflict of interest, including any funding they received for conducting the review? | Yes | Yes | Yes | Yes | Yes | Yes | Yes | Yes |
| **Overall risk of bias** | **High** | **Low** | **High** | **High** | **Moderate** | **Low** | **Moderate** | **Moderate** |

*Abbreviations: NA=not applicable*

**Table S9:** Extraction tables on the primary study level

1. **The original and the adjusted Gail models/Breast Cancer Risk Assessment Tool**

| Study ID [reference]/ included in SR [reference] | Country of application of the model | Female  population | Risk model | | | | |
| --- | --- | --- | --- | --- | --- | --- | --- |
|  |  |  | Model version | Sample size | Predicting breast cancer type/risk trajectory | Included  risk factors | Discriminatory accuracy:  AUC (95% CI) |
| **Gail 1989 [2]**/Louro 2019 [23], Al-Ajmi 2018 [21], Stegeman 2012 [20], Anothaisintawee 2012 [18] | USA | Caucasian aged 20-79 yrs | Original Gail model^a^ | 5,998 | Invasive and in situ bc/ probabilities within 10, 20, 30 yrs of follow-up | Age, menarche, previous biopsies, age at first birth, first-degree bc family history | - |
| **Spiegelman 1994 [35]**/ Stegeman 2012 [20], Meads 2012 [19], Wang 2018 [22] | USA | n.r. aged 29-61 yrs | Gail 1^b^ | 115,172 | Invasive and  in situ bc/5 yrs | Age, menarche, previous biopsies, age at first birth, first-degree bc family history | - |
| **Bondy 1994 [36]**/ Meads 2012 [19], Wang 2018 [22] | USA | White (high-risk) aged 30–75 yrs | Gail 1^c^ | 1,981 | Invasive and  in situ bc/5 yrs | Age, menarche, previous biopsies, age at first birth, first-degree bc family history |  |
| **Costantino 1999 [37]**/ Stegeman 2012 [20], Meads 2012 [19], Wang 2018 [22] | USA | White aged > 35 yrs | Gail 2^d^ NCI model | 5,969 | Invasive bc/5 yrs | Age, menarche, previous biopsies, age at first birth, first-degree bc family history | - |
|  |  |  | Gail 1 |  | Invasive and in situ bc/5 yrs |  |  |
| **Rockhill 2001 [38]**/ Stegeman 2012 [20], Meads 2012 [19], Wang 2018 [22] | USA | White aged 45-71 yrs | Gail 2 | 82,109 | Invasive bc/5 yrs | Age, menarche, previous biopsies, age at first birth, first-degree bc family history | 0.58 (0.56-0.60) |
| **Amir 2003 [39]**/ Meads 2012 [19], Wang 2018 [22] | UK | European aged 21-73 yrs | Gail 2 | 3,150 | Invasive bc/10 yrs | Age, menarche, previous biopsies, age at first birth, first-degree bc family history | 0.74 (0.667-0.80) |
| **Olson 2004 [40]**/ Wang 2018 [22] | USA | women with bilateral oophorectomy aged 31-90 yrs | Gail 1 | 674 | Invasive and  in situ bc/5 yrs | n.r. | - |
| **Bernatsky 2004 [41]**/ Wang 2018 [22] | USA | Mixed ethnic (high-risk) mean age 41 yrs | Gail 1 | 871 | Invasive and  in situ bc/5 yrs | n.r. | - |
| **Boyle 2004 [42]**/ Louro 2019 [23], Anothaisintawee 2012 [18], Meads 2012 [19], Wang 2018 [22] | Italy | European aged 20–74 yrs | Gail 2 | 5,383 | Invasive bc/5 yrs | Age, menarche, age at first birth, first-degree bc, *BMI, alcohol, physical activity, HRT, diet beta-carotene/vitE, diet fruits/ vegetables* | 0.582 |
| **Tice 2005 [43]**/ Louro 2019 [23], Meads 2012 [19], Anothaisintawee 2012 [18], Wang 2018 [22] | USA | Mixed ethnic  aged > 35 yrs | Modified Gail^e^ | 81,777 | Invasive and  in situ bc/n.r. | Age, menarche, previous biopsies, age at first birth, first-degree bc family history, *breast density* | 0.67 (0.65–0.68) |
| **Tice 2005 [44]**/ Anothaisintawee 2012 [18] | USA | Mixed ethnic age not specified | Modified Gail | 6,904 | Invasive bc/n.r. | Age, menarche, previous biopsies, age at first birth, first-degree bc family history, *nipple aspirate fluid cytology* | 0.64 |
| **Chen 2006 [10]**/ Louro 2019 [23], Anothaisintawee 2012 [18], Meads 2012 [19] | USA | Caucasian aged 35-74 yrs | Gail 2 | 5,315 | Invasive and  in situ bc/n.r. | Age, menarche, age at first birth, first-degree bc family history, HRT, previous biopsies, BMI, *atypical hyperplasia, breast density* | 0.602 |
| **Novotny 2006 [45]**/ Louro 2019 [23], Al-Ajmi 2018 [21], Anothaisintawee 2012 [18], Meads 2012 [19] | Czech Republic | European  aged 23-84 yrs | Modified Gail | 4,598 | Invasive bc/n.r. | Age, menarche, previous biopsies, age at first birth, first-degree bc family history, *parity, breast inflammatory disease* | - |
| **Decarli 2006 [46]**/ Louro 2019 [23], Stegeman 2012 [20], Anothaisintawee 2012 [18], Meads 2012 [19], Wang 2018 [22] | Italy | European  aged 35-64 yrs | Gail 2 | 10,031 | Invasive bc/5 yrs | Age, menarche, previous biopsies, age at first birth, first-degree bc family history | 0.59 (0.55–0.63) |
|  |  |  |  |  |  |  |  |
| **Gail 2007 [47]**/ Louro 2019 [23], Al-Ajmi 2018 [21], Stegeman 2012 [20], Anothaisintawee 2012 [18], Meads 2012 [19] | USA | African-American  aged 35-64 yrs | CARE model^f^ | 3,254 | Invasive bc/n.r. | Age, menarche, previous biopsies, age at first birth, first-degree bc family history | 0.56 (0.535-0.575) |
| **Adams-Campbell 2007 [48]**/ Stegeman 2012 [20] | USA | African-American aged >35 yrs | Modified Gail | 1,450 | n.r. | Age, menarche, previous biopsies, age at first birth, first-degree bc family history, *atypical hyperplasia* | close to 0.5 |
| **Chlebowski 2007 [49]**/ Stegeman 2012 [20], Anothaisintawee 2012 [18], Wang 2018 [22] | USA | Mixed ethnic aged 50-79 yrs | Modified Gail | 151,152 | Invasive bc/5 yrs | Age, previous biopsies, first degree  bc family history, *parity, breast feed­ing, smoking, alcohol, BMI, physical activity, hormone replacement therapy* | 0.58 (0.56-0.60) |
| **Crispo 2008 [50]**/ Wang 2018 [22] | Italy | European mean age 53.7 yrs | Gail 1 | 1,765 | Invasive and in situ bc/5 yrs | n.r. | 0.55 (0.53-0.58) |
| **Tice 2008 [51]**/ Wang 2018 [22] | USA | Mixed ethnic aged 40-74 yrs | Gail 2 | 629,229 | Invasive bc/5 yrs | n.r. | 0.61 (0.60-0.62) |
| **Pan [52]**/ Wang 2018 [22] | China | Chinese aged > 35 yrs | Gail 1 | 2,133 | Invasive and  in situ bc/5 yrs | n.r. | 0.64 (0.61-0.67) |
| **Liu 2010 [53]**/ Wang 2018 [22] | China | Chinese mean age 49.8 yrs | Gail 2 | 246 | Invasive bc/5 yrs | n.r. | 0.56 (0.49-0.64) |
| **Wang 2010 [54]**/ Wang 2018 [22] | China | Chinese aged 32-75 yrs | Gail 1 | 228 | Invasive and  in situ bc/5 yrs | n.r. | 0.93 (0.89-0.97) |
| **Schonfeld 2010 [55]**/ Meads 2012 [19], Wang 2018 [22] | USA | White post-menopausal mean age 62,8 yrs | Gail 2 | 181,979 NIH-AARP | Invasive bc/5 yrs | n.r. | - |
|  |  | White post-menopausal mean age 62,3 yrs |  | 64,868 PLCO |  |  | - |
| **Mealiffe 2010 [56]**/Fung 2019 [24] | USA | White (post-menopausal) aged 50-79 yrs | Modified Gail | 3,300 | Invasive bc/n.r. | Age, menarche, previous biopsies, age at first birth, first-degree bc family history, *ethnicity,  polygenic risk score (7)* | *without* *SNPs:*  0.56 (0.54-0.57)  *SNP-enhanced:* 0.59 (0.57-0.61) ¥ |
| **Wacholder 2010 [57]**/ Fung 2019 [24], Meads 2012 [19] | USA, Poland | European aged 50-79 yrs | Modified Gail | 11,588 | Invasive bc/n.r. | Age, menarche, previous biopsies, age at first birth, first-degree bc family history, *polygenic risk score (10)* | *without SNPs:* 0.58  *SNP-enhanced:* 0.62 |
| **Tarabishy 2011 [58]**/ Wang 2018 [22] | USA | American (high-risk) aged 18-85 yrs | Gail 2 | 4,726 | Invasive bc/5 yrs | n.r. | 0.64 (0.62-0.66) |
| **Vacek 2011 [59]**/ Wang 2018 [22] | USA | American (high-risk) aged > 70 yrs | Gail 1 | 19,779 | Invasive and  in situ bc/5 yrs | n.r. | 0.54 (0.52-0.56) |
| **Matsuno 2011 [60]**/ Louro 2019 [23], Al-Ajmi 2018 [21] | USA | Asian  aged 20-55 yrs | Modified  Gail | 1,541 | Invasive bc/n.r. | Age, menarche, previous biopsies, age at first birth, first-degree bc family history, *ethnicity* | 0.61 |
| **Quante [61]**/ Wang 2018 [22] | USA | American (high-risk) mean age 44 yrs | Gail 2 | 1,857 | Invasive bc/10 yrs | n.r. | 0.63 (0.58-0.69) |
| **Banegas 2012 [62]**/ Al-Ajmi 2018 [21], Wang 2018 [22] | USA | Hispanic  mean age 63.51 yrs | Gail 2 | 128,976 | Invasive bc/5 yrs | Age, menarche, previous biopsies, age at first birth, first-degree bc family history | 0.58 (0.57-0.59) |
| **Darabi 2012 [63]**/ Vilmun 2020 [25], Fung 2019 [24] | Sweden | European (post-menopausal) aged 5074 yrs | Modified Gail | 1,890 | n.r./5 yrs | Age, menarche, previous biopsies, age at first birth, first-degree bc family history, *atypical hyperplasia, BMI, breast density^2^, ethnicity, polygenic risk score (18)* | *without density:* 0.57 (0.55-0.59)  *PD% :*0.60 (0.58-0.62) |
|  |  |  |  |  |  |  | *without SNPs:* 0.57 (0.54-0.597)  *SNP-enhanced:* 0.62 (0.59-0.64)¥ |
|  |  |  |  | 3,093 |  | Age, menarche, age at first birth, bc family history, benign breast disease | *without SNPs:*  0.55 (0.53-0.57)  *SNP-enhanced: 0.62 (0.59-0.63)* ¥ |
| **Higginbotham 2012 [64]**/ Fung 2019 [24] | USA | Caucasian aged 20-74 yrs | Modified Gail | 2,737 | Invasive or in situ bc/n.r. | Age, menarche, age at first birth, bc family history, *biopsy histo­pathology, benign breast surgery* | *without SNPs:* 0.588  *SNP-enhanced:* 0.615 |
|  |  | Caucasian aged 16-96 yrs |  | 1,760 |  | Age, menarche, previous biopsies, age at first birth, first-degree bc family history, *biopsy histo­pathology, polygenic risk score (9)* | *without SNPs:* 0.573  *SNP-enhanced:* 0.601 |
| **Maclnnis 2012 [65]**/ Wang 2018 [22] | Australia | Female relatives of bc cases in Australia (high risk) age not specified | n.r. | 2,000 | n.r. | n.r. | *-* |
| **Chay 2012 [66]**/ Wang 2018 [22] | Singapore | Asian aged 50–64 yrs | Gail 3^g^ | 28,104 | Invasive or in situ bc/5 yrs | n.r. | - |
|  |  |  |  |  | Invasive or in situ bc/10 yrs |  |  |
| **Pastor-Barriuso 2013 [67]**/ Wang 2018 [22] | Spain | European aged 45–68 yrs | Gail 2 | 54,649 | Invasive bc/5 yrs | n.r. | 0.54 (0.52-0.57) |
| **Buron 2013 [68]**/ Wang 2018 [22] | Spain | European (high-risk) aged 49–64 yrs | Gail 2 | 2,200 | Invasive bc/5 yrs | n.r. | *-* |
| **Park 2013 [69]**/ Al-Ajmi 2018 [21] | Korea | Korean-Asian  any age | Modified Gail | 7,578 | Invasive bc/n.r. | Age, menarche, previous biopsies, age at first birth, bc family history, *menopausal status, number of pregnancies, duration of breast­feeding, oral contraceptive usage, exercise, smoking, drinking, and number of breast examinations* | 0.65 |
| **Anothaisintawee 2013 [70]**/ Wang 2018 [22] | Thailand | Asian age not specified | n.r. | 15,718 | n.r. | n.r. | 0.41 (0.36-0.46) |
| **Rosner 2013 [7]**/ Wang 2018 [22] | USA | American (high-risk) age not specified | Gail 2 | 11,419 | Invasive bc/5 yrs | n.r. | 0.55 (0.53-0.56) |
| **Dite 2013 [71]**/ Fung 2019 [24], Wang 2018 [22] | Australia | Australian overall | Modified Gail | 1,425 | Invasive bc/n.r. | Age, menarche, previous biopsies, age at first birth, first-degree bc family history, *ethnicity, polygenic risk score (7)* | *Overall without SNPs:  0.58 (0.55-0.61)*  *SNP-enhanced: 0.61 (0.58-0.64)* |
|  |  | aged 35-39 yrs |  |  |  |  | *without SNPs:* 0.60 (0.55-0.60)  *SNP-enhanced:* 0.65 (0.60-0.700) |
|  |  | aged 40-49 yrs |  |  |  |  | *without SNPs:* 0.61 (0.55-0.66)  *SNP-enhanced:* 0.63 (0.57-0.69) |
|  |  | aged 50-59 yrs |  |  |  |  | *without SNPs:* 0.54 (0.48-0.60)  *SNP-enhanced:* 0.56 (0.51-0.62) |
| **Min 2014 [72]**/ Wang 2018 [22] | Korea | Asian aged > 10 yrs | Gail 2 | 40,229 | Invasive bc/5 yrs | n.r. | 0.55 (0.50-0.59) |
|  |  |  | Gail 3 |  |  |  | 0.54 (0.50-0.59) |
| **Duan 2014 [73]**/ Wang 2018 [22] | China | Asian aged 35-74 yrs | Gail 2 | 400 | Invasive bc/5 yrs | n.r. | 0.54 (0.49-0.60) |
| **Jupe 2014 [74]**/ Fung 2019 [24] | USA | Caucasian aged 35-39 yrs | Modified Gail | 5,022 | n.r. | Age, menarche, age at first birth, first-degree bc family history, *polygenic risk score (22)* | *without SNPs:* 0.580  *SNP-enhanced:* 0.690¥ |
| **Powell 2014 [75]**/ Wang 2018 [22] | USA | American (high-risk) age not specified | Gail 2 | 12,843 | Invasive bc/5 yrs | n.r. | 0.62 (0.59-0.66) |
| **Allman 2015 [76]**/ Fung 2019 [24] | USA | African-American aged >49 yrs | Modified Gail | 7,421 | n.r. | Age, menarche, age at first birth, first-degree bc family history, *polygenic risk score (75)* | *without SNPs:* 0.56 (0.53-0.59)  *SNP-enhanced:* 0.59 (0.56-0.61) |
|  |  | Hispanic aged >49 yrs |  | 3,348 |  | Age, menarche, age at first birth, first-degree bc family history, *polygenic risk score (71)* | *without SNPs:* 0.55 (0.51-0.60)  *SNP-enhanced:* 0.61 (0.56-0.66) |
| **Wu 2015 [77]**/ Fung 2019 [24] | USA | European aged 29-90 yrs | Modified Gail | 768 | Invasive or in situ bc/n.r. | Age, menarche, previous biopsies, first-degree bc family history,  *age at biopsy, parity, 5 BI-RADS mammographic features, polygenic risk score (10)* | *without SNPs:* 0.597  *SNP-enhanced:* 0.638¥ |
| **McCarthy 2015 [78]**/ Wang 2018 [22] | USA | American (high-risk) aged 35-62 yrs | Gail 2 | 464 | Invasive bc/5 yrs | n.r. | 0.71 (0.65-0.78) |
| **Dartois 2015 [79]**/ Wang 2018 [22] | France | European aged 42–72 yrs | Gail 2 | 13,174 | Invasive bc/5 yrs | n.r. | - |
|  |  | Pre-menopausal aged 42–72 yrs |  | 5,843 |  |  | 0.61 (0.55-0.68) |
|  |  | Post-menopausal  (high risk) aged 42–72 yrs |  | 7,331 |  |  | 0.55 (0.50-0.60) |
| **Hu 2015 [80]**/ Wang 2018 [22] | China | Chinese aged 35-69 yrs | Gail 2 | 42,908 | Invasive bc/5 yrs | n.r. | 0.59 (0.47-0.70) |
| **Schonberg 2015 [81]**/ Wang 2018 [22] | USA | American (high-risk) aged 63-77 yrs | Gail 2 | 71,293 | Invasive bc/5 yrs | n.r. | 0.57 (0.55-0.58) |
|  |  |  |  | 79,611 |  |  | 0.58 (0.56-0.59) |
| **Keller 2015 [82]**/ Vilmun 2020 [25] | USA | Caucasian aged >40 yrs | Modified Gail | 424 | Invasive bc/n.r. | Age, menarche, previous biopsies, age at first birth, first-degree bc family history, atypical *breast density^3^, ethnicity* | *without density* 0.64 (0.58-0.71)  *PD% ^4^:* 0.68 (0.62-0.74)  *PD% + absolute dense area+ absolute dense tissue volume + (VD%) 0.77 (0.71-0.82)* |
|  |  |  |  |  |  | Age, menarche, previous biopsies, age at first birth, first-degree bc family history, *breast density^3^, ethnicity, BMI* | *without density:* 0.8 (0.75-0.85)  *PD% ^3^:* 0.85 (0.81-0.90)  *PD% + absolute dense area+ absolute dense tissue volume + (VD% ): 0.86 (0.82-0.90)* |
| **Lee 2015 [83]**/ Al-Ajmi 2018 [21] | Korea | Asian any age | Modified Gail | 4,574 | Invasive bc/n.r. | Age, menarche, age at first birth,  bc family history, *number of children, BMI, menopausal status, regular mammography, regular exercise* | 0.62 |
| **Brentnall 2015 [84]**/ Vilmun 2020 [25], Wang 2018 [22] | UK | European aged 47–73 yrs | Modified Gail | 50,628 | Invasive and  in situ bc/10 yrs | Age, menarche, previous biopsies, age at first birth, bc family history, *breast density^1^, ethnicity* | *without density:* Invasive + DCIS 0.55 (0.52-0.62) Invasive:  0.54 (0.52-0.56)  *With density:* Invasive + DCIS:  0.59 (0.57-0.61) Invasive: 0.59  (0.57-0.61) |
| **Lee 2015 [85]**/ Vilmun 2020 [25], Fung 2019 [24] | Singapore | Asian aged 50–64 yrs | Modified Gail | 24,161 | Invasive and  in situ bc/10 yrs | Age, menarche, previous biopsies, age at first birth, first-degree bc family history, *BMI, breast density3, ethnicity, polygenic  risk score (75)* | *without density:* 0.63 (0.61-0.65)  *PD% ^4^:* 0.65 (0.63-0.67)  *absolute dense area*:  0.66 (0.64-0.68)  *without SNPs:* 0.66 (0.64-0.680)  *SNP-enhanced*: 0.68 (0.66-0.69) |
| **Rong 2016 [86]**/ Wang 2018 [22] | China | Asian mean age 48.9 yrs | Gail 1 | 816 | Invasive and  in situ bc/5 yrs | n.r. | 0.69 (0.68-0.71) |
| **Dite 2016 [87]**/ Fung 2019 [24] | Australia | Caucasian (non-BRCA mutation carriers) aged <50 yrs | Gail 2 | 1,155 | Invasive bc/n.r. | Age, menarche, first-degree bc family history (biopsies or atypical hyperplasia not available), *polygenic risk score (77)* | *without SNPs:* 0.640 (0.600-0.680)  *SNP-enhanced:* 0.67 (0.63-0.700)¥ |
| **Banegas 2017 [88]**/ Louro 2019 [23] | USA | Hispanic  aged 25-79 yrs | Gail 1 | 2,497 | Invasive bc/projected absolute risk within 5, 10, 20, 30 yrs | Age, menarche, previous biopsies, age at first birth, first-degree bc family history | 0.56 US-born 0.62 foreign-born |
| **Zhang 2018 [89]**/ Louro 2019 [23] | USA | Caucasian  aged 30-64 yrs | Modified Gail | 11,880 | Invasive bc/ 5 yrs | Age, menarche, previous biopsies, age at first birth, first-degree bc family history, *mammographic breast density, polygenic risk score, estrone sulfate,* *testosterone, prolactin* | 0.65 |

Abbreviations: AUC=area under the curve, bc=breast cancer, DCIS=ductal carcinoma in situ, CI=confidence interval, HRT=hormone replacement therapy, n.r.=not reported, NCI=National Cancer Institute, NIH-AARP=National Institutes of Health (NIH) –AARP Diet and Health Study, PD%=area percent density, PLCO=Prostate, Lung, Colorectal and Ovarian Cancer Screening Trial, SEER=Surveillance, Epidemiology, and End Results database, SNPs=single nucleotide polymorphisms, SR=systematic review, UK=United Kingdom, USA=United States of America, VD%=volume percent density, yrs=years

^a^ Age-specific incidence rates based on Breast Cancer Detection and Demonstration Project (BCDDP).

^b^ Validated in the Nurses' Health Study.

^c^ Validated in Texas Breast Screening Project (cohort of women with a family history of breast cancer).

^d^ Gail Model 2: incidence rates include only invasive cancers, age-specific incidence rates obtained from the Surveillance, Epidemiology and End Results database, composite incidence rates for African-Americans.

^e^ Modified Gail Model includes race/ethnicity and/or other risk factors.

^f^ CARE model estimates risk in black women.

^g^ Classification according to Wang [22] Gail model 3=modified Gail model for Asian-American.

^1^ Breast density visually assessed.

^2^ Breast density semi-automated Cumulus method [90].

^3^ Breast density fully automated (absolute dense area and area percent density estimated using a publicly available software tool [91]. Absolute dense tissue volume and volume percent density are automatically assessed by using FDA-cleared software Quantra™ version 2.0).

^¥^ The SNP-enhanced model showed a statistically significant improvement (p<0.05) over the model without SNPs.

^£^ Split SEER cohort by date from 1983–1987 and 1995-2003.

1. **The original and the adapted Breast Cancer Surveillance Consortium models**

| Study ID [reference]/ included in SR [reference] | Country of application of the model | Female  population | Risk model | | | | |
| --- | --- | --- | --- | --- | --- | --- | --- |
|  |  |  | Model  ID | Sample size | Predicting breast cancer type/risk trajectory | Included  risk factors | Discriminatory accuracy: AUC (95% CI) |
| **Tice 2008 [51]**/ Louro 2019 [23], Meads 2012 [19] | USA | Mixed ethnic  aged 35-84 yrs | Tice model/ BCSC model | 1,095,484 | Invasive bc/n.r. | Age, ethnicity, first-degree bc family history, previous biopsies, breast density (BIRADS) | 0.66 (0.651-0.669) |
| **Kerlikowske 2015 [92]**/, Louro 2019 [23] | USA | Mixed ethnic  aged 35-74 yrs | BCSC model | 722,654 | Invasive bc/5 yrs and 10 yrs bc risk | Age, ethnicity, first-degree bc family history, previous biopsies*, changes in breast density* | 0.64 |
| **Tice 2015 [5]**/ Louro 2019 [23] | USA | Mixed ethnic  aged 35-74 yrs | BCSC model | 1,135,977 | Invasive bc/5 yrs and 10 yrs bc risk | Age, ethnicity, first-degree bc family history, previous biopsies, breast density, *benign breast disease* | 0.67 |
| **Vachon 2015 [93]**/ Louro 2019 [23], Fung 2019 [24] | USA | Mixed ethnic  aged 35-74 yrs | BCSC model v1.0 | 4,040 | Invasive bc/n.r. | Age, ethnicity, first-degree bc family history, previous biopsies, breast density (BIRADS), *polygenic risk score (76)* | *without SNPs:* 0.660 (0.610-0.700)  *SNP enhanced:* 0.69 (0.670-0.710) ^¥^ |
| **Shieh 2016 [94]**/ Louro 2019 [23], Fung 2019 [24] | USA | Mixed ethnic aged 36-86 yrs | BCSC model v2.0 | 931 | Invasive bc/n.r. | Age, ethnicity, first-degree bc family history, previous biopsies, breast density (BI-RADS), *polygenic risk sore (83), BMI* | *without SNPs:* 0.620 (0.520-0.730)  *SNP enhanced:* 0.650 (0.610-0.680) ^¥^ |
| **Shieh 2017 [95]**/ Fung 2019 [24] | USA | Mixed ethnic (post-menopausal) | BCSC model v1.0 | 324 | ER-positive bc/n.r. | Age, ethnicity, first-degree bc family history, previous biopsies, breast density (BI-RADS), polygenic risk score (83), BMI | *without SNPs:* 0.580 (0.500-0.650)  *SNP enhanced:* 0.640 (0.570-0.710) |
|  |  |  |  |  |  | Age, ethnicity, first-degree bc family history, previous biopsies, breast density (BI-RADS), polygenic risk score, BMI, estradiol | *without SNPs:* 0.670 (0.600-0.740)  *SNP enhanced:* 0.720 (0.650-0.790)^¥^ |

*Abbreviations: AUC=area under the curve, bc=breast cancer, BMI=body mass index, CI=confidence interval, DCIS=ductal carcinoma in situ, ER=estrogen receptor, MHT=menopausal hormone therapy, n.a.=not applicable, n.r.=not reported, PD%=Percent dense area, SNPs=single-nucleotide polymorphisms, VD%=volume percent density, USA=United States of America, yrs=years*

*^¥^ The SNP-enhanced model showed a statistically signiﬁcant improvement (P < 0.05) over the model without SNPs.*

1. **The original and the adapted Rosner and Colditz models**

| Study ID [reference]/ included in SR [reference] | Country of application of the model | Female population | Risk model | | | | |
| --- | --- | --- | --- | --- | --- | --- | --- |
|  |  |  | Model | Sample size | Predicting breast cancer type/risk trajectory | Included  risk factors | Discriminatory accuracy:  AUC (95% CI) |
| **Rosner 1994 [96]**/ Al-Ajmi 2018 [21], Meads 2012 [19] | USA | Caucasian aged 30-55 yrs | Previous model | 93,864 | n.r. | Age, menarche, age at first birth, menopause, age at subsequent births | - |
| **Rosner 1996 [6]**/ Louro 2019 [23], Al-Ajmi 2018 [21], Stegeman 2012 [20], Anothaisintawee 2012 [18], Meads 2012 [19] | USA | Caucasian aged 30-64 yrs | Original | 89,132 | Invasive bc/n.r. | Age, menarche, age at first birth, menopause, age at subsequent births, *no. of breast biopsies* | - |
| **Colditz 2000 [97]**/ Louro 2019 [23], Al-Ajmi 2018 [21], Stegeman 2012 [20], Anothaisintawee 2012 [18], Meads 2012 [19] | USA | Caucasian aged 30-64 yrs | Modified Rosner and Colditz model | 58,520 | Invasive bc/n.r. | Age, menarche, age at first birth, menopause, age at subsequent births, *no. of breast biopsies,* *benign breast disease,* *HRT, first-degree bc family history, weight, BMI, alcohol* | - |
| **Rockhill 2003 [98]**/ Anothaisintawee 2012 [18], Meads 2012 [19], Stegeman 2012 [20] | USA | Caucasian age not specified | Original | 45,210 | Invasive bc/n.r. | Age, menarche, age at first birth, menopause, age at subsequent births, *no. of breast biopsies* | 0.57 (0.55–0.59) |
|  |  |  | Modified Rosner and Colditz model |  |  | Age, menarche, age at first birth, menopause, age at subsequent births, *no. of breast biopsies, benign breast disease,* *HRT, first-degree bc family history, weight, BMI, alcohol* | 0.64 (0.62–0.66) |
| **Colditz 2004 [99]**/ Louro 2019 [23], Anothaisintawee 2012 [18] | USA | Caucasian  aged 30-64 yrs | Modified Rosner and Colditz model | 66,145 | Invasive bc/n.r. | Age, menarche, age at first birth, menopause, age at subsequent births, *no. of breast biopsies, benign breast disease, HRT, first-degree bc family history, weight, BMI, alcohol* | 0.64 ER+/PR+ 0.61 ER-/PR- |
| **Rosner 2008 [100]**/ Louro 2019 [23], Anothaisintawee 2012 [18], Meads 2012 [19] | USA | Caucasian  aged 30-64 yrs | Modified Rosner and Colditz model | 59,812 | Hormonal defined bc/n.r. | Age, menarche, age at first birth, menopause, age at subsequent births, *benign breast disease, HRT, first-degree bc family history, weight, BMI, alcohol, estradiol levels* | 0.635 (0.628–0.642) |
| **Zhang 2008 [89]**/ Louro 2019 [23] | USA | Caucasian  aged 30-64 yrs | Modified Rosner and Colditz model | 11,880 | Invasive bc/n.r. | Age, menarche, age at first birth, menopause, age at subsequent births, *HRT, first-degree bc family history, weight, BMI, alcohol, early life somatotype, polygenic risk score, mammographic breast density, estrone sulfate, testosterone, prolactin* | 0.68 |
| **Tamimi 2010 [101]**/ Anothaisintawee 2012 [18] | USA | Caucasian age not specified | Modified Rosner and Colditz model | 78,243 | Invasive bc/n.r. | Age, menarche, age at first birth, menopause, age at subsequent births, *HRT, first-degree bc family history, weight, BMI, alcohol, type of benign breast disease (atypical hyperplasia)* | 0.64 |
| **Viallon 2009 [102]**/ Meads 2012 [19] | France | European age not specified | Original | 91,968 | Invasive bc/n.r. | Age, menarche, age at first birth, menopause, age at subsequent births | - |

*Abbreviations: AUC=area under the curve, BC=breast cancer, BMI=body mass index, CI=confidence interval, HRT=hormone replacement therapy, n.r.=not reported, SR=systematic review, USA=United States of America, yrs=years*

1. **The original and the adapted International Breast Cancer Intervention Study models**

| Study ID [reference]/ included in SR [reference] | Country of application of the model | Female  population | Risk model | | | | |
| --- | --- | --- | --- | --- | --- | --- | --- |
|  |  |  | Model | Sample  size | Predicting breast cancer type/risk trajectory | Included  risk factors | Discriminatory accuracy:  AUC (95% CI) |
| **Tyrer 2004 [15]**/ Louro 2019 [23], Meads 2012 [19] | UK | Mixed ethnic age not specified | Original | n.r. | Invasive bc/n.r. | Age, family history (relationship, age), menarche, age at first birth, menopause, atypical hyperplasia, LCIS, height, BMI | - |
| **Amir 2003 [39]**/ Meads 2012 [19] | UK | European (high-risk) age not specified | Tyrer-Cuzick model | 3,150 | n.r. | n.r. | 0.762 (0.700–0.824) |
| **Warwick 2014 [103]**/ Vilmun 2020 [25] | UK | n.r. (high risk) aged 35-70 yrs | Tyrer-Cuzick model | 558 | Invasive bc and DCIS/10 yrs | Age, BMI, age at menarche, age at first live birth, parital status, menopausal status, previous biopsy, HRT usage, atypical hyperplasia/ LCIS, *breast density** | *without density*: 0.51  with density: 0.62 |
| **Van Veen 2018 [104]**/ Vilmun 2020 [25], Fung 2019 [24] | UK | European aged 46-73 yrs | Tyrer-Cuzick model | 9,363 | Invasive bc and DCIS/10 yrs | Age, BMI, age at menarche, age at first live birth, age at menopause, parital status, HRT usage, previous biopsy, history of atypical hyperplasia, *ethnicity, breast density*, polygenic risk score (18)* | *without density*: 0.58 (0.52−0.62)  *with density:* 0.64 (0.60−0.68) |
|  |  |  |  |  |  |  | *without SNPs:* 0.640 (0.600-0.680)  *SNP-enhanced:* 0.670 (0.620–0.710) |
| **Brentnall 2015 [84]**/ Vilmun 2020 [25] | UK | Europan aged 47-73 yrs | Tyrer-Cuzick model | 50,628 | Invasive bc and DCIS/10 yrs | Age, BMI, age at menarche, age at first live birth, age at menopause, parital status, HRT usage, previous biopsy, history of atypical hyperplasia, *ethnicity, breast density** | *without density:*  invasive + DCIS 0.57 (0.55−0.59)  invasive 0.57 (0.55−0.59) |
|  |  |  |  |  |  |  | *with density:*  invasive + DCIS 0.61 (0.59−0.63)  invasive 0.61 (0.58−0.63) |
| **Brentnall 2018 [105]**/ Vilmun 2020 [25] | USA | American aged 40-73 yrs | Tyrer-Cuzick model | 132,139 | Invasive bc/10 yrs | Age, BMI, age at menarche, age at first live birth, menopausal status, parital status, HRT usage, previous biopsy, history of atypical hyperplasia, *ethnicity, breast density** | - |
| **Allman 2015 [76]**/ Fung 2019 [24] | USA | African-American | Tyrer-Cuzick model | 7,421 | Invasive bc/5 yrs | Age, menarche, age at first birth, menopause, LCIS, height, BMI, no information on family history and atypical hyperplasia, *polygenic risk score (>70)* | *without SNPs:* 0.51 (0.48-0.54)  *SNP-enhanced:* 0.55 (0.52–0.58) |
|  |  | Hispanic |  | 3,348 |  |  | *without SNPs:* 0.53 (0.48-0.57)  *SNP-enhanced:* 0.59 (0.54–0.64) |
| **Dite 2016 [87]**/ Fung 2019 [24] | Australia | Caucasian <50 yrs (non-BRCA mutation carriers) age not specified | IBIS model | 1,155 | Invasive bc/n.r. | Age, family history (relationship, age), menarche, age at first birth, menopause, height, BMI, hyperplasia or LCIS information not available), *polygenic risk score (77)* | *without SNPs:* 0.57 (0.53–0.60)  *SNP-enhanced:* 0.63 (0.59–0.66) ^¥^ |

Abbreviations: AUC=area under the curve, bc=breast cancer, BMI=body mass index, CI=confidence interval, DCIS=ductal carcinoma in situ, HRT=hormone replacement therapy, LCIS=lobular carcinoma in situ, n.r.=not reported, SNPs=single-nucleotide polymorphisms, SR=systematic review, UK=United Kingdom, USA=United States of America, yrs=years

^*^ Visually assessed.

© Family history of the woman is used to calculate the distribution of her genotype probabilities, and the phenotypic probabilities are calculated.

^¥^ The SNP-enhanced model showed a statistically significant improvement (p<0.05) over the model without SNPs.

1. **The BOADICEA and BRCAPRO™ models**

| Study ID [reference]/ included in SR [Reference] | Country of application of the model | Female  population | Risk model | | | | |
| --- | --- | --- | --- | --- | --- | --- | --- |
|  |  |  | Model  ID | Sample size | Predicting breast cancer type/risk trajectory | Included  risk factors | Discriminatory accuracy: AUC (95% CI) |
| **Dite 2016 [87]**/ Fung 2019 [24] | Australia | Caucasian <50 yrs (non-BRCA mutation carriers) | BOADICEA model | 1,155 | Invasive bc/n.r. | Age, family history of bc  (1^st^, 2^nd^, 3^rd^ degree relatives, age at  bc cancer diagnosis, bilateral bc, male bc, ovarian cancer), polygenic risk score (77) | *without SNPs:* 0.660 (0.630-0.700)  *SNP-enhanced:* 0.700 (0.670-0.730) ^¥^ |
| **Dite 2016 [87]**/ Fung 2019 [24] | Australia | Caucasian <50 yrs (non-BRCA mutation carriers) | BRCAPRO™ model | 1,155 | Invasive bc/n.r. | Age, ethnicity, family history of bc  (1^st^, 2^nd^, 3^rd^ degree relatives, age at  bc cancer diagnosis, bilateral bc, male bc, ovarian cancer), polygenic risk score (77) | *without SNPs:* 0.650 (0.620-0.680)  *SNP-enhanced:* 0.690 (0.660-0.720) ^¥^ |

*Abbreviations: AUC=area under the curve, bc=breast cancer, CI=confidence interval, n.r.=not reported, SNPs=single-nucleotide polymorphisms, yrs=years*

*^¥^ The SNP-enhanced model showed a statistically significant improvement (p<0.05) over the model without SNPs.*

1. **Further original models**

| Study ID [reference]/ included in SR [reference] | Country of application of the model | Female  population | Risk model | | | | |
| --- | --- | --- | --- | --- | --- | --- | --- |
|  |  |  | Model | Sample  size | Predicting breast cancer type/risk trajectory | Included  risk factors | Discriminatory accuracy: AUC (95% CI) |
| **Barlow 2006 [3]**/ Louro 2019 [23], Anothaisintawee 2012 [18], Meads 2012 [19] | USA | Mixed ethnic  aged 35-84 yrs | Original | 1,007,600 | Invasive bc and in situ carcinomas/n.r. | Age, age at first birth, ethnicity, menopause, first-degree bc family history, previous biopsies, breast density, HRT, BMI, previous false positive screen result | 0.631 pre-menopausal status  0.624 post-menopausal status |
| **Eriksson 2017 [106]**/ Louro 2019 [23] | Sweden | Caucasian aged 40-74 yrs | Original | 2,165 | Invasive bc and in situ carcinomas/n.r. | Age, BMI, HRT, bc family history, menopause, breast density, microcalcifications, masses | 0.71 |
| **Ueda 2003 [107]**/ Louro 2019 [23], Al-Ajmi 2018 [21], Anothaisintawee 2012 [18] | Japan | Asian age not specified | Original | 806 | Invasive bc/n.r. | Age, menarche, age at first birth, BMI,  bc family history | - |
| **Wang 2014 [108]**/ Louro 2019 [23] | China | Asian aged 35-70 yrs | Original | n.a. | Invasive bc/n.r. | Age, menarche, previous biopsies, age at first birth, first-degree bc family history, breastfeeding, abortion | 0.64 |
| **Pfeiffer 2013 [109]**/ Al-Ajmi 2018 [21] | USA | White/ non-Hispanic Caucasian  aged > 50 yrs | Original | 240,712 | Invasive bc/n.r. | BMI, oestrogen and progestin MHT use, other MHT use, parity, age at first birth, menopausal status, age at menopause, benign breast diseases, family history of breast or ovarian cancer, alcohol consumption | 0.58 |
| **Lee 2004 [110]**/ Al-Ajmi 2018 [21], Anothaisintawee 2012 [18] | Korea | Korean-Asian aged ≥ 20 yrs | Original | 550 | Invasive bc/n.r. | Age, age at menarche, age at meno­pause, age at first live birth, family history of bc, breastfeeding, alcohol, smoking | - |
| **Abdolell 2016 [111]**/ Vilmun 2020 [25] | Canada | Canadian aged 40-75 yrs | Original | 1,209 | n.r. | Age, number of births, HRT usage, first-degree relatives with bc, menopausal status, breast density^1^ | *without density:* 0.54 (0.50−0.57)  *PD% + absolute dense area+non-dense area:* 0.63 (0.60−0.67) |
| **Saikiran 2019 [112]**/ Vilmun 2020 [25] | India | Indian mean age 50.2 yrs | Original | 650 | Invasive bc/n.r. | Age, BMI, number of births, HRT usage, family history of bc, menopausal status, breast density^1^ | *without density:* 0.58 (0.45−0.64)  *PD%:* 0.72 (0.64−0.80) |
| **Shepherd 2011 [113]**/ Vilmun 2020 [25] | USA | American mean age 57.1 yrs | Original | 1,100 | Invasive bc and DCIS/n.r. | BMI, age at first live birth, first-degree relatives with bc, history of atypical hyperplasia, breast density^1,2^ | *without density*: 0.61  *absolute dense volume:* 0.65  *VD%:* 0.65 |
| **Häberle 2012 [114]**/ Vilmun 2020 [25] | Germany | European mean age 57 yrs | Original | 1,282 | n.r. | Age, BMI, parital status, family history of bc, age at first term pregnancy, breast density^2^ | *without density*: 0.65  *PD%:* 0.66 |
| **Rauh 2012 [115]**/ Vilmun 2020 [25] | Germany | European age not specified | Original | 937 | Invasive bc/n.r. | Age, BMI, parital status, HRT usage, menopausal status, breast density^2^ | *without density*: 0.65  *absolute dense area*: 0.65  *combined densities:* 0.68 |
| **Zheng 2010 [116]**/ Fung 2019 [24] | China | Chinese age not specified | Original | 6,121 | Invasive bc/n.r. | Age, age at menarche, age at first live birth, waist-to-hip ratio, benign breast disease, family history of bc, BMI, parity, *polygenic risk score (8)* | *without SNPs:* 0.618  *SNP-enhanced:* 0.630 |
| **Kaklamani 2011 [117]**/ Fung 2019 [24] | USA | Mixed ethnic age not specified | Original | 718 | Invasive bc/n.r. | Age, race, BMI,  *polygenic risk score (4)* | *without SNPs:* 0.530  *SNP-enhanced:* 0.600 |
| **Dai 2012 [118]**/ Fung 2019 [24] | China | Chinese age not specified | Original | 1,881 | n.r. | Age at first live birth, age at menarche, *polygenic risk score (5)* | *without SNPs:* 0.638  *SNP-enhanced:* 0.649 |
| **Hüsing 2012 [119]**/ Fung 2019 [24] | USA | European age not specified | Original | 13,836 | Invasive bc/n.r. | BMI, BMI interaction with menopausal status, age at ﬁrst parity, age at menopause, no.of full-term pregnancies, use of HRT, age at menarche, alcohol consumption, smoking status, polygenic risk score (7,9,18,32) | *without SNPs:* 0.564 (0.547-0.581)  *SNP-enhanced:  7 SNPs:* 0.591(0.574-0.608)  *9 SNPs:* 0.595 (0.579-0.612)  *18 SNPs:* 0.605 (0.589-0.622)^¥^  *32 SNPs:* 0.604 (0.588-0.621) |
| **Sueta 2012 [120]**/ Fung 2019 [24] | Japan | Japanese age not specified | Original | 2,091 | n.r. | Age, age at menarche, age at first live birth, menopausal status, family history of breast cancer, BMI, regular exercise, referral pattern to hospital, polygenic risk score (7) | *without SNPs:* 0.665  *SNP-enhanced:* 0.693 |
| **Xu 2013 [121]**/ Fung 2019 [24] | USA | white women who have had a biological sister with bc age not specified | Original | 910 | n.r. | Age at menarche, age at first live birth, no. of biopsies, no. of first-degree relatives with bc, polygenic risk score (9) | *without SNPs:* 0.560  *SNP-enhanced:* 0.612 |
| **Lee 2014 [122]**/ Fung 2019 [24] | Singapore | Chinese age not specified | Original | 1,623 | n.r. | Level of education, age at first live birth, age at menarche, history of past breast biopsy, family history, BMI, polygenic risk score (51) | *-* |
| **Wen 2016 [123]**/ Fung 2019 [24] | USA | East Asian age not specified | Original | 23,567 | n.r. | Age at menarche, age at first live birth, waist-to-hip ratio, prior benign breast disease (yes/no), family history of bc (yes/no), polygenic risk score (44) | *without SNPs:* 0.563  *SNP enhanced:* 0.602 |
| **Burnside [124]**/ Fung 2019 [24] | USA | White (non-BRCA mutation carriers) age not specified | Original | 768 | Invasive or in situ bc/n.r. | Age, age at menarche, no. of first-degree relatives with bc, previous biopsies, parity, polygenic risk score (10) | *without SNPs:* 0.547 (0.50-0.587)  *SNP enhanced:* 0.601 (0.562-0.641) |
| **Maas 2016 [125]**/ Fung 2019 [24] | USA | White age not specified | Original | 37,033 | Invasive bc/n.r. | Family history, age at first live birth, parity, age at menarche, height, menopausal status, age at menopause, BMI, menopausal hormone therapy use, level of alcohol consumption, smoking status, polygenic risk score (92) | *without SNPs: 0.588*  *SNP enhanced: 0.648* |
| **Hsieh 2017 [126]**/ Fung 2019 [24] | Taiwan | Asian age not specified | Original | 960 | Invasive or in situ bc/n.r. | Age, BMI, age at menarche, parity, menopausal status, polygenic risk score (13) | *without SNPs:* 0.634  *SNP-enhanced:* 0.665 |
| **Guo 2017 [127]**/ Fung 2019 [24] | Japan | Japanese pre-menopausal age not specified | Original | 406 | ER-positive, HER2-negative, invasive and non-invasive cancers/n.r. | Age, BMI, age at menarche, age at menopause (post-menopausal), no. of pregnancy, parity (yes/no), age at primiparity, breastfeeding, benign breast disease (yes/no), family history of breast cancer (yes/no), hyperlipidemia (yes/no), diabetes mellitus (yes/no), testosterone serum levels, 25-hydroxyvitamin D serum levels, polygenic risk score  (4 pre-menopausal, 2 post-menopausal) | *without SNPs:* 0.708  *SNP-enhanced:* 0.785 |
|  |  | Japanese post-menopausal age not specified |  | 752 |  |  | *without SNPs:* 0.693  *SNP-enhanced:* 0.764 |
| **Arne 2009 [128]**/ Meads 2012 [19] | Mazedonien | n.r. | n.r. | n.r. | n.r. | Age, birth history/parity, condom use, family history of bc, reproductive age period | *-* |

Abbreviations: AUC=area under the curve, bc=breast cancer, BMI=body mass index, CI=confidence interval, DCIS=ductal carcinoma in situ, ER=estrogen receptor, MHT=menopausal hormone therapy, n.a.=not applicable, n.r.=not reported, PD%=Percent dense area, SNPs=single-nucleotide polymorphisms, SR=systematic review, VD%=volume percent density, USA=United States of America, yrs=years

^1^ Fully automated

^2^ Semi-automated

^¥^ The SNP-enhanced model showed a statistically signiﬁcant improvement (P < 0.05) over the model without SNPs.

**References**

[1] Pruthi S, Heisey R, Bevers T. Personalized assessment and management of women at risk for breast cancer in North America. Womens Health (Lond). 2015;11(2):213-23; 23-4.

[2] Gail MH, Brinton LA, Byar DP, Corle DK, Green SB, Schairer C, et al. Projecting individualized probabilities of developing breast cancer for white females who are being examined annually. J Natl Cancer Inst. 1989;81(24):1879-86.

[3] Barlow WE, White E, Ballard-Barbash R, Vacek PM, Titus-Ernstoff L, Carney PA, et al. Prospective breast cancer risk prediction model for women undergoing screening mammography. J Natl Cancer Inst. 2006;98(17):1204-14.

[4] Breast Cancer Surveillance Consortium Risk Calculator [cited 18 July 2022]. Available from: <http://tools.bcsc-scc.org/BC5yearRisk/>.

[5] Tice JA, Miglioretti DL, Li CS, Vachon CM, Gard CC, Kerlikowske K. Breast Density and Benign Breast Disease: Risk Assessment to Identify Women at High Risk of Breast Cancer. J Clin Oncol. 2015;33(28):3137-43.

[6] Rosner B, Colditz GA. Nurses' health study: log-incidence mathematical model of breast cancer incidence. J Natl Cancer Inst. 1996;88(6):359-64.

[7] Rosner BA, Colditz GA, Hankinson SE, Sullivan-Halley J, Lacey JV, Jr., Bernstein L. Validation of Rosner-Colditz breast cancer incidence model using an independent data set, the California Teachers Study. Breast Cancer Res Treat. 2013;142(1):187-202.

[8] Colditz GA, Atwood KA, Emmons K, Monson RR, Willett WC, Trichopoulos D, et al. Harvard report on cancer prevention volume 4: Harvard Cancer Risk Index. Risk Index Working Group, Harvard Center for Cancer Prevention. Cancer Causes Control. 2000;11(6):477-88.

[9] Glynn RJ, Colditz GA, Tamimi RM, Chen WY, Hankinson SE, Willett WW, et al. Extensions of the Rosner-Colditz breast cancer prediction model to include older women and type-specific predicted risk. Breast Cancer Res Treat. 2017;165(1):215-23.

[10] Chen J, Pee D, Ayyagari R, Graubard B, Schairer C, Byrne C, et al. Projecting absolute invasive breast cancer risk in white women with a model that includes mammographic density. J Natl Cancer Inst. 2006;98(17):1215-26.

[11] McClintock AH, Golob AL, Laya MB. Breast Cancer Risk Assessment: A Step-Wise Approach for Primary Care Providers on the Front Lines of Shared Decision Making. Mayo Clin Proc. 2020;95(6):1268-75.

[12] Antoniou AC, Pharoah PD, McMullan G, Day NE, Stratton MR, Peto J, et al. A comprehensive model for familial breast cancer incorporating BRCA1, BRCA2 and other genes. Br J Cancer. 2002;86(1):76-83.

[13] Antoniou AC, Pharoah PP, Smith P, Easton DF. The BOADICEA model of genetic susceptibility to breast and ovarian cancer. Br J Cancer. 2004;91(8):1580-90.

[14] Parmigiani G, Berry D, Aguilar O. Determining carrier probabilities for breast cancer-susceptibility genes BRCA1 and BRCA2. Am J Hum Genet. 1998;62(1):145-58.

[15] Tyrer J, Duffy SW, Cuzick J. A breast cancer prediction model incorporating familial and personal risk factors. Stat Med. 2004;23(7):1111-30.

[16] Tyrer-Cuzick/IBIS Risk Assessment Tool v8.0b: Wolfson Institute of Preventive Medicine, Queen Mary University of London. 2022 [cited 18 July 2022]. Available from: <https://ibis.ikonopedia.com/>.

[17] Claus EB, Risch N, Thompson WD. Autosomal dominant inheritance of early-onset breast cancer. Implications for risk prediction. Cancer. 1994;73(3):643-51.

[18] Anothaisintawee T, Teerawattananon Y, Wiratkapun C, Kasamesup V, Thakkinstian A. Risk prediction models of breast cancer: a systematic review of model performances. Breast Cancer Res Treat. 2012;133(1):1-10.

[19] Meads C, Ahmed I, Riley RD. A systematic review of breast cancer incidence risk prediction models with meta-analysis of their performance. Breast Cancer Res Treat. 2012;132(2):365-77.

[20] Stegeman I, Bossuyt PM. Cancer risk models and preselection for screening. Cancer Epidemiol. 2012;36(5):461-9.

[21] Al-Ajmi K, Lophatananon A, Yuille M, Ollier W, Muir KR. Review of non-clinical risk models to aid prevention of breast cancer. Cancer Causes Control. 2018;29(10):967-86.

[22] Wang X, Huang Y, Li L, Dai H, Song F, Chen K. Assessment of performance of the Gail model for predicting breast cancer risk: a systematic review and meta-analysis with trial sequential analysis. Breast Cancer Res. 2018;20(1):18.

[23] Louro J, Posso M, Hilton Boon M, Román M, Domingo L, Castells X, et al. A systematic review and quality assessment of individualised breast cancer risk prediction models. Br J Cancer. 2019;121(1):76-85.

[24] Fung SM, Wong XY, Lee SX, Miao H, Hartman M, Wee HL. Performance of Single-Nucleotide Polymorphisms in Breast Cancer Risk Prediction Models: A Systematic Review and Meta-analysis. Cancer Epidemiol Biomarkers Prev. 2019;28(3):506-21.

[25] Vilmun BM, Vejborg I, Lynge E, Lillholm M, Nielsen M, Nielsen MB, et al. Impact of adding breast density to breast cancer risk models: A systematic review. Eur J Radiol. 2020;127:109019.

[26] WANFANG database. [cited 18 July 2022]. Available from: <http://www.wanfangdata.com.cn/>.

[27] VIP database. [cited 18 July 2022]. Available from: <http://www.cqvip.com/>.

[28] China National Knowledge Infrastructure database. [cited 18 July 2022]. Available from: <http://www.cnki.net/>.

[29] Altman DG, Vergouwe Y, Royston P, Moons KG. Prognosis and prognostic research: validating a prognostic model. Bmj. 2009;338:b605.

[30] Stang A. Critical evaluation of the Newcastle-Ottawa scale for the assessment of the quality of nonrandomized studies in meta-analyses. Eur J Epidemiol. 2010;25(9):603-5.

[31] Whiting P, Rutjes AW, Reitsma JB, Bossuyt PM, Kleijnen J. The development of QUADAS: a tool for the quality assessment of studies of diagnostic accuracy included in systematic reviews. BMC Med Res Methodol. 2003;3:25.

[32] Jaime Caro J, Eddy DM, Kan H, Kaltz C, Patel B, Eldessouki R, et al. Questionnaire to assess relevance and credibility of modeling studies for informing health care decision making: an ISPOR-AMCP-NPC Good Practice Task Force report. Value Health. 2014;17(2):174-82.

[33] Janssens AC, Ioannidis JP, Bedrosian S, Boffetta P, Dolan SM, Dowling N, et al. Strengthening the reporting of genetic risk prediction studies (GRIPS): explanation and elaboration. Eur J Hum Genet. 2011;19(5):18 p preceding 494.

[34] Wells GA, Shea B, O'Connell D, Peterson J, Welch V, Losos M, et al. The Newcastle-Ottawa Scale (NOS) for assessing the quality of nonrandomised studies in meta-analyses [cited 18 July 2022]. Available from: <http://www.ohri.ca/programs/clinical_epidemiology/oxford.asp>.

[35] Spiegelman D, Colditz GA, Hunter D, Hertzmark E. Validation of the Gail et al. model for predicting individual breast cancer risk. J Natl Cancer Inst. 1994;86(8):600-7.

[36] Bondy ML, Lustbader ED, Halabi S, Ross E, Vogel VG. Validation of a breast cancer risk assessment model in women with a positive family history. J Natl Cancer Inst. 1994;86(8):620-5.

[37] Costantino JP, Gail MH, Pee D, Anderson S, Redmond CK, Benichou J, et al. Validation studies for models projecting the risk of invasive and total breast cancer incidence. J Natl Cancer Inst. 1999;91(18):1541-8.

[38] Rockhill B, Spiegelman D, Byrne C, Hunter DJ, Colditz GA. Validation of the Gail et al. model of breast cancer risk prediction and implications for chemoprevention. J Natl Cancer Inst. 2001;93(5):358-66.

[39] Amir E, Evans DG, Shenton A, Lalloo F, Moran A, Boggis C, et al. Evaluation of breast cancer risk assessment packages in the family history evaluation and screening programme. J Med Genet. 2003;40(11):807-14.

[40] Olson JE, Sellers TA, Iturria SJ, Hartmann LC. Bilateral oophorectomy and breast cancer risk reduction among women with a family history. Cancer Detect Prev. 2004;28(5):357-60.

[41] Bernatsky S, Clarke A, Ramsey-Goldman R, Joseph L, Boivin JF, Rajan R, et al. Hormonal exposures and breast cancer in a sample of women with systemic lupus erythematosus. Rheumatology (Oxford). 2004;43(9):1178-81.

[42] Boyle P, Mezzetti M, La Vecchia C, Franceschi S, Decarli A, Robertson C. Contribution of three components to individual cancer risk predicting breast cancer risk in Italy. Eur J Cancer Prev. 2004;13(3):183-91.

[43] Tice JA, Cummings SR, Ziv E, Kerlikowske K. Mammographic breast density and the Gail model for breast cancer risk prediction in a screening population. Breast Cancer Res Treat. 2005;94(2):115-22.

[44] Tice JA, Miike R, Adduci K, Petrakis NL, King E, Wrensch MR. Nipple aspirate fluid cytology and the Gail model for breast cancer risk assessment in a screening population. Cancer Epidemiol Biomarkers Prev. 2005;14(2):324-8.

[45] Novotny J, Pecen L, Petruzelka L, Svobodnik A, Dusek L, Danes J, et al. Breast cancer risk assessment in the Czech female population--an adjustment of the original Gail model. Breast Cancer Res Treat. 2006;95(1):29-35.

[46] Decarli A, Calza S, Masala G, Specchia C, Palli D, Gail MH. Gail model for prediction of absolute risk of invasive breast cancer: independent evaluation in the Florence-European Prospective Investigation Into Cancer and Nutrition cohort. J Natl Cancer Inst. 2006;98(23):1686-93.

[47] Gail MH, Costantino JP, Pee D, Bondy M, Newman L, Selvan M, et al. Projecting individualized absolute invasive breast cancer risk in African American women. J Natl Cancer Inst. 2007;99(23):1782-92.

[48] Adams-Campbell LL, Makambi KH, Palmer JR, Rosenberg L. Diagnostic accuracy of the Gail model in the Black Women's Health Study. Breast J. 2007;13(4):332-6.

[49] Chlebowski RT, Anderson GL, Lane DS, Aragaki AK, Rohan T, Yasmeen S, et al. Predicting risk of breast cancer in post-menopausal women by hormone receptor status. J Natl Cancer Inst. 2007;99(22):1695-705.

[50] Crispo A, D'Aiuto G, De Marco M, Rinaldo M, Grimaldi M, Capasso I, et al. Gail model risk factors: impact of adding an extended family history for breast cancer. Breast J. 2008;14(3):221-7.

[51] Tice JA, Cummings SR, Smith-Bindman R, Ichikawa L, Barlow WE, Kerlikowske K. Using clinical factors and mammographic breast density to estimate breast cancer risk: development and validation of a new predictive model. Ann Intern Med. 2008;148(5):337-47.

[52] Pan XP, Jin X, Ding H. Preliminary study on risk evaluation model of breast cancer in Beijing and Guangdong. Matern Child Health Care of China. 2009;11:1469–71.

[53] Liu LY. A pilot study on risk factors and risk assessment score screening model for high-risk population of breast cancer. Shandong University 2010.

[54] Wang Y, Xu L, Shen CJ, et al. Clinical application of Gail model in the assessment of breast cancer risk. Int J Pathol Clin Med. 2010;6:473–5.

[55] Schonfeld SJ, Pee D, Greenlee RT, Hartge P, Lacey JV, Jr., Park Y, et al. Effect of changing breast cancer incidence rates on the calibration of the Gail model. J Clin Oncol. 2010;28(14):2411-7.

[56] Mealiffe ME, Stokowski RP, Rhees BK, Prentice RL, Pettinger M, Hinds DA. Assessment of clinical validity of a breast cancer risk model combining genetic and clinical information. J Natl Cancer Inst. 2010;102(21):1618-27.

[57] Wacholder S, Hartge P, Prentice R, Garcia-Closas M, Feigelson HS, Diver WR, et al. Performance of common genetic variants in breast-cancer risk models. N Engl J Med. 2010;362(11):986-93.

[58] Tarabishy Y, Hartmann LC, Frost MH, Maloney SD, Vierkant RA, Pankratz VS. Performance of the Gail model in individual women with benign breast disease. Journal of Clinical Oncology. 2011;29:1525.

[59] Vacek PM, Skelly JM, Geller BM. Breast cancer risk assessment in women aged 70 and older. Breast Cancer Res Treat. 2011;130(1):291-9.

[60] Matsuno RK, Costantino JP, Ziegler RG, Anderson GL, Li H, Pee D, et al. Projecting individualized absolute invasive breast cancer risk in Asian and Pacific Islander American women. J Natl Cancer Inst. 2011;103(12):951-61.

[61] Quante AS, Whittemore AS, Shriver T, Strauch K, Terry MB. Breast cancer risk assessment across the risk continuum: genetic and nongenetic risk factors contributing to differential model performance. Breast Cancer Res. 2012;14(6):R144.

[62] Banegas MP, Gail MH, LaCroix A, Thompson B, Martinez ME, Wactawski-Wende J, et al. Evaluating breast cancer risk projections for Hispanic women. Breast Cancer Res Treat. 2012;132(1):347-53.

[63] Darabi H, Czene K, Zhao W, Liu J, Hall P, Humphreys K. Breast cancer risk prediction and individualised screening based on common genetic variation and breast density measurement. Breast Cancer Res. 2012;14(1):R25.

[64] Higginbotham KS, Breyer JP, McReynolds KM, Bradley KM, Schuyler PA, Plummer WD, et al. A multistage genetic association study identifies breast cancer risk loci at 10q25 and 16q24. Cancer Epidemiol Biomarkers Prev. 2012;21(9):1565-73.

[65] MacInnis R, Dite G, Bickerstaffe A, Dowty J, Aujard K, Apicella C, et al. Validation study of risk prediction models for female relatives of Australian women with breast cancer. Hereditary Cancer in Clinical Practice. 2012;10(2):A66.

[66] Chay WY, Ong WS, Tan PH, Jie Leo NQ, Ho GH, Wong CS, et al. Validation of the Gail model for predicting individual breast cancer risk in a prospective nationwide study of 28,104 Singapore women. Breast Cancer Res. 2012;14(1):R19.

[67] Pastor-Barriuso R, Ascunce N, Ederra M, Erdozáin N, Murillo A, Alés-Martínez JE, et al. Recalibration of the Gail model for predicting invasive breast cancer risk in Spanish women: a population-based cohort study. Breast Cancer Res Treat. 2013;138(1):249-59.

[68] Buron A, Vernet M, Roman M, Checa MA, Pérez JM, Sala M, et al. Can the Gail model increase the predictive value of a positive mammogram in a European population screening setting? Results from a Spanish cohort. Breast. 2013;22(1):83-8.

[69] Park B, Ma SH, Shin A, Chang MC, Choi JY, Kim S, et al. Korean risk assessment model for breast cancer risk prediction. PLoS One. 2013;8(10):e76736.

[70] Anothaisintawee T, Thakkinstian A, Wiratkapun C. Developing and validating risk prediction model for screening breast cancer in Thai women. Eur J Epidemiol 2013;28 (Suppl 1):96–7.

[71] Dite GS, Mahmoodi M, Bickerstaffe A, Hammet F, Macinnis RJ, Tsimiklis H, et al. Using SNP genotypes to improve the discrimination of a simple breast cancer risk prediction model. Breast Cancer Res Treat. 2013;139(3):887-96.

[72] Min JW, Chang MC, Lee HK, Hur MH, Noh DY, Yoon JH, et al. Validation of risk assessment models for predicting the incidence of breast cancer in korean women. J Breast Cancer. 2014;17(3):226-35.

[73] Duan XK, Luo ZY, Chen L, et al. The application of the Gail breast cancer prediction model in Chinese women. Matern Child Health Care of China. 2014;28:4667–9.

[74] Jupe ER, Dalessandri KM, Mulvihill JJ, Miike R, Knowlton NS, Pugh TW, et al. A steroid metabolizing gene variant in a polyfactorial model improves risk prediction in a high incidence breast cancer population. BBA Clin. 2014;2:94-102.

[75] Powell M, Jamshidian F, Cheyne K, Nititham J, Prebil LA, Ereman R. Assessing breast cancer risk models in Marin County, a population with high rates of delayed childbirth. Clin Breast Cancer. 2014;14(3):212-20.e1.

[76] Allman R, Dite GS, Hopper JL, Gordon O, Starlard-Davenport A, Chlebowski R, et al. SNPs and breast cancer risk prediction for African American and Hispanic women. Breast Cancer Res Treat. 2015;154(3):583-9.

[77] Wu Y, Liu J, Del Rio AM, Page DC, Alagoz O, Peissig P, et al. Developing a clinical utility framework to evaluate prediction models in radiogenomics. Proc SPIE Int Soc Opt Eng. 2015;9416.

[78] McCarthy AM, Keller B, Kontos D, Boghossian L, McGuire E, Bristol M, et al. The use of the Gail model, body mass index and SNPs to predict breast cancer among women with abnormal (BI-RADS 4) mammograms. Breast Cancer Res. 2015;17(1):1.

[79] Dartois L, Gauthier É, Heitzmann J, Baglietto L, Michiels S, Mesrine S, et al. A comparison between different prediction models for invasive breast cancer occurrence in the French E3N cohort. Breast Cancer Res Treat. 2015;150(2):415-26.

[80] Hu JY. Research on the applicability of Gail model in the assessment of breast cancer risk in Zhejiang eastern coastal women. Zhejiang University Medical College; 2015.

[81] Schonberg MA, Li VW, Eliassen AH, Davis RB, LaCroix AZ, McCarthy EP, et al. Performance of the Breast Cancer Risk Assessment Tool Among Women Age 75 Years and Older. J Natl Cancer Inst. 2016;108(3).

[82] Keller BM, Chen J, Daye D, Conant EF, Kontos D. Preliminary evaluation of the publicly available Laboratory for Breast Radiodensity Assessment (LIBRA) software tool: comparison of fully automated area and volumetric density measures in a case-control study with digital mammography. Breast Cancer Res. 2015;17:117.

[83] Lee C, Lee JC, Park B, Bae J, Lim MH, Kang D, et al. Computational Discrimination of Breast Cancer for Korean Women Based on Epidemiologic Data Only. J Korean Med Sci. 2015;30(8):1025-34.

[84] Brentnall AR, Harkness EF, Astley SM, Donnelly LS, Stavrinos P, Sampson S, et al. Mammographic density adds accuracy to both the Tyrer-Cuzick and Gail breast cancer risk models in a prospective UK screening cohort. Breast Cancer Res. 2015;17(1):147.

[85] Lee CP, Choi H, Soo KC, Tan MH, Chay WY, Chia KS, et al. Mammographic Breast Density and Common Genetic Variants in Breast Cancer Risk Prediction. PLoS One. 2015;10(9):e0136650.

[86] Rong L, Li H, Wang EL. To establish the breast cancer risk prediction model for women in Shenzhen in China Matern Child Health Care of China. 2016;3:470–3.

[87] Dite GS, MacInnis RJ, Bickerstaffe A, Dowty JG, Allman R, Apicella C, et al. Breast Cancer Risk Prediction Using Clinical Models and 77 Independent Risk-Associated SNPs for Women Aged Under 50 Years: Australian Breast Cancer Family Registry. Cancer Epidemiol Biomarkers Prev. 2016;25(2):359-65.

[88] Banegas MP, John EM, Slattery ML, Gomez SL, Yu M, LaCroix AZ, et al. Projecting Individualized Absolute Invasive Breast Cancer Risk in US Hispanic Women. J Natl Cancer Inst. 2017;109(2).

[89] Zhang X, Rice M, Tworoger SS, Rosner BA, Eliassen AH, Tamimi RM, et al. Addition of a polygenic risk score, mammographic density, and endogenous hormones to existing breast cancer risk prediction models: A nested case-control study. PLoS Med. 2018;15(9):e1002644.

[90] Byng JW, Boyd NF, Fishell E, Jong RA, Yaffe MJ. The quantitative analysis of mammographic densities. Phys Med Biol. 1994;39(10):1629-38.

[91] Center for Biomedical Image Computing and Analytics. Laboratory for Individualized Breast Radiodensity Assessment (LIBRA) homepage. [cited 18 July 2022]. Available from: <https://www.cbica.upenn.edu/sbia/software/LIBRA/index.html>.

[92] Kerlikowske K, Zhu W, Tosteson AN, Sprague BL, Tice JA, Lehman CD, et al. Identifying women with dense breasts at high risk for interval cancer: a cohort study. Ann Intern Med. 2015;162(10):673-81.

[93] Vachon CM, Pankratz VS, Scott CG, Haeberle L, Ziv E, Jensen MR, et al. The contributions of breast density and common genetic variation to breast cancer risk. J Natl Cancer Inst. 2015;107(5).

[94] Shieh Y, Hu D, Ma L, Huntsman S, Gard CC, Leung JW, et al. Breast cancer risk prediction using a clinical risk model and polygenic risk score. Breast Cancer Res Treat. 2016;159(3):513-25.

[95] Shieh Y, Hu D, Ma L, Huntsman S, Gard CC, Leung JWT, et al. Joint relative risks for estrogen receptor-positive breast cancer from a clinical model, polygenic risk score, and sex hormones. Breast Cancer Res Treat. 2017;166(2):603-12.

[96] Rosner B, Colditz GA, Willett WC. Reproductive risk factors in a prospective study of breast cancer: the Nurses' Health Study. Am J Epidemiol. 1994;139(8):819-35.

[97] Colditz GA, Rosner B. Cumulative risk of breast cancer to age 70 years according to risk factor status: data from the Nurses' Health Study. Am J Epidemiol. 2000;152(10):950-64.

[98] Rockhill B, Byrne C, Rosner B, Louie MM, Colditz G. Breast cancer risk prediction with a log-incidence model: evaluation of accuracy. J Clin Epidemiol. 2003;56(9):856-61.

[99] Colditz GA, Rosner BA, Chen WY, Holmes MD, Hankinson SE. Risk factors for breast cancer according to estrogen and progesterone receptor status. J Natl Cancer Inst. 2004;96(3):218-28.

[100] Rosner B, Colditz GA, Iglehart JD, Hankinson SE. Risk prediction models with incomplete data with application to prediction of estrogen receptor-positive breast cancer: prospective data from the Nurses' Health Study. Breast Cancer Res. 2008;10(4):R55.

[101] Tamimi RM, Rosner B, Colditz GA. Evaluation of a breast cancer risk prediction model expanded to include category of prior benign breast disease lesion. Cancer. 2010;116(21):4944-53.

[102] Viallon V, Ragusa S, Clavel-Chapelon F, Bénichou J. How to evaluate the calibration of a disease risk prediction tool. Stat Med. 2009;28(6):901-16.

[103] Warwick J, Birke H, Stone J, Warren RM, Pinney E, Brentnall AR, et al. Mammographic breast density refines Tyrer-Cuzick estimates of breast cancer risk in high-risk women: findings from the placebo arm of the International Breast Cancer Intervention Study I. Breast Cancer Res. 2014;16(5):451.

[104] van Veen EM, Brentnall AR, Byers H, Harkness EF, Astley SM, Sampson S, et al. Use of Single-Nucleotide Polymorphisms and Mammographic Density Plus Classic Risk Factors for Breast Cancer Risk Prediction. JAMA Oncol. 2018;4(4):476-82.

[105] Brentnall AR, Cuzick J, Buist DSM, Bowles EJA. Long-term Accuracy of Breast Cancer Risk Assessment Combining Classic Risk Factors and Breast Density. JAMA Oncol. 2018;4(9):e180174.

[106] Eriksson M, Czene K, Pawitan Y, Leifland K, Darabi H, Hall P. A clinical model for identifying the short-term risk of breast cancer. Breast Cancer Res. 2017;19(1):29.

[107] Ueda K, Tsukuma H, Tanaka H, Ajiki W, Oshima A. Estimation of individualized probabilities of developing breast cancer for Japanese women. Breast Cancer. 2003;10(1):54-62.

[108] Wang Y, Gao Y, Battsend M, Chen K, Lu W, Wang Y. Development of a risk assessment tool for projecting individualized probabilities of developing breast cancer for Chinese women. Tumour Biol. 2014;35(11):10861-9.

[109] Pfeiffer RM, Park Y, Kreimer AR, Lacey JV, Jr., Pee D, Greenlee RT, et al. Risk prediction for breast, endometrial, and ovarian cancer in white women aged 50 y or older: derivation and validation from population-based cohort studies. PLoS Med. 2013;10(7):e1001492.

[110] Lee EO, Ahn SH, You C, Lee DS, Han W, Choe KJ, et al. Determining the main risk factors and high-risk groups of breast cancer using a predictive model for breast cancer risk assessment in South Korea. Cancer Nurs. 2004;27(5):400-6.

[111] Abdolell M, Tsuruda KM, Lightfoot CB, Payne JI, Caines JS, Iles SE. Utility of relative and absolute measures of mammographic density vs clinical risk factors in evaluating breast cancer risk at time of screening mammography. Br J Radiol. 2016;89(1059):20150522.

[112] Saikiran P, Ramzan R, S N, Kamineni PD, Priyanka, John AM. Mammographic Breast Density Assessed with Fully Automated Method and its Risk for Breast Cancer. J Clin Imaging Sci. 2019;9:43.

[113] Shepherd JA, Kerlikowske K, Ma L, Duewer F, Fan B, Wang J, et al. Volume of mammographic density and risk of breast cancer. Cancer Epidemiol Biomarkers Prev. 2011;20(7):1473-82.

[114] Häberle L, Wagner F, Fasching PA, Jud SM, Heusinger K, Loehberg CR, et al. Characterizing mammographic images by using generic texture features. Breast Cancer Res. 2012;14(2):R59.

[115] Rauh C, Hack CC, Häberle L, Hein A, Engel A, Schrauder MG, et al. Percent Mammographic Density and Dense Area as Risk Factors for Breast Cancer. Geburtshilfe Frauenheilkd. 2012;72(8):727-33.

[116] Zheng W, Wen W, Gao YT, Shyr Y, Zheng Y, Long J, et al. Genetic and clinical predictors for breast cancer risk assessment and stratification among Chinese women. J Natl Cancer Inst. 2010;102(13):972-81.

[117] Kaklamani V, Yi N, Sadim M, Siziopikou K, Zhang K, Xu Y, et al. The role of the fat mass and obesity associated gene (FTO) in breast cancer risk. BMC Med Genet. 2011;12:52.

[118] Dai J, Hu Z, Jiang Y, Shen H, Dong J, Ma H, et al. Breast cancer risk assessment with five independent genetic variants and two risk factors in Chinese women. Breast Cancer Res. 2012;14(1):R17.

[119] Hüsing A, Canzian F, Beckmann L, Garcia-Closas M, Diver WR, Thun MJ, et al. Prediction of breast cancer risk by genetic risk factors, overall and by hormone receptor status. J Med Genet. 2012;49(9):601-8.

[120] Sueta A, Ito H, Kawase T, Hirose K, Hosono S, Yatabe Y, et al. A genetic risk predictor for breast cancer using a combination of low-penetrance polymorphisms in a Japanese population. Breast Cancer Res Treat. 2012;132(2):711-21.

[121] Xu Z, Bolick SC, DeRoo LA, Weinberg CR, Sandler DP, Taylor JA. Epigenome-wide association study of breast cancer using prospectively collected sister study samples. J Natl Cancer Inst. 2013;105(10):694-700.

[122] Lee CP, Irwanto A, Salim A, Yuan JM, Liu J, Koh WP, et al. Breast cancer risk assessment using genetic variants and risk factors in a Singapore Chinese population. Breast Cancer Res. 2014;16(3):R64.

[123] Wen W, Shu XO, Guo X, Cai Q, Long J, Bolla MK, et al. Prediction of breast cancer risk based on common genetic variants in women of East Asian ancestry. Breast Cancer Res. 2016;18(1):124.

[124] Burnside ES, Liu J, Wu Y, Onitilo AA, McCarty CA, Page CD, et al. Comparing Mammography Abnormality Features to Genetic Variants in the Prediction of Breast Cancer in Women Recommended for Breast Biopsy. Acad Radiol. 2016;23(1):62-9.

[125] Maas P, Barrdahl M, Joshi AD, Auer PL, Gaudet MM, Milne RL, et al. Breast Cancer Risk From Modifiable and Nonmodifiable Risk Factors Among White Women in the United States. JAMA Oncol. 2016;2(10):1295-302.

[126] Hsieh YC, Tu SH, Su CT, Cho EC, Wu CH, Hsieh MC, et al. A polygenic risk score for breast cancer risk in a Taiwanese population. Breast Cancer Res Treat. 2017;163(1):131-8.

[127] Guo J, Sueta A, Nakamura K, Yoshimoto N, Baba M, Ishida N, et al. Genetic and environmental factors and serum hormones, and risk of estrogen receptor-positive breast cancer in pre- and post-menopausal Japanese women. Oncotarget. 2017;8(39):65759-69.

[128] Arne GN. Breast cancer risk assessments to barrier contraception exposure. A new approach. Makedonska Akademija na Naukite i Umetnostite Oddelenie Za Bioloshki i Meditsinski Nauki Prilozi 2009;30(1):217–32.
